# PIWIL1 governs the crosstalk of cancer cell metabolism and immunosuppressive microenvironment in hepatocellular carcinoma

**DOI:** 10.1038/s41392-021-00485-8

**Published:** 2021-02-26

**Authors:** Ning Wang, Hor-Yue Tan, Yuanjun Lu, Yau-Tuen Chan, Di Wang, Wei Guo, Yu Xu, Cheng Zhang, Feiyu Chen, Guoyi Tang, Yibin Feng

**Affiliations:** 1 School of Chinese Medicine, Li Ka Shing Faculty of Medicine, The University of Hong Kong, Hong Kong SAR, People’s Republic of China; 2 School of Life Sciences, Jilin University, Changchun City, Jilin Province People’s Republic of China

**Keywords:** Gastrointestinal cancer, Cancer microenvironment

## Abstract

Altered energy metabolism of cancer cells shapes the immune cell response in the tumor microenvironment that facilitates tumor progression. Herein, we reported the novel of tumor cell-expressed Piwi Like RNA-Mediated Gene Silencing 1 (PIWIL1) in mediating the crosstalk of fatty acid metabolism and immune response of human hepatocellular carcinoma (HCC). PIWIL1 expression in HCC was increased compared to normal hepatic tissues and was positively correlated with the proliferation rate of HCC cell lines. PIWIL1 overexpression accelerated in vitro proliferation and in vivo growth of HCC tumors, while PIWIL1 knockdown showed opposite effects. PIWIL1 increased oxygen consumption and energy production via fatty acid metabolism without altering aerobic glycolysis. Inhibition of fatty acid metabolism abolished PIWIL1-induced HCC proliferation and growth. RNA-seq analysis revealed that immune system regulation might be involved, which was echoed by the experimental observation that PIWIL1-overexpressing HCC cells attracted myeloid-derived suppressor cells (MDSCs) into the tumor microenvironment. MDSCs depletion reduced the proliferation and growth of PIWIL1-overexpressing HCC tumors. Complement C3, whose secretion was induced by PIWIL1 in HCC cells, mediates the interaction of HCC cells with MDSCs by activated p38 MAPK signaling in MDSCs, which in turn initiated expression of immunosuppressive cytokine IL10. Neutralizing IL10 secretion reduced the immunosuppressive activity of MDSCs in the microenvironment of PIWIL1-overexpressing HCC. Taken together, our study unraveled the critical role of PIWIL1 in initiating the interaction of cancer cell metabolism and immune cell response in HCC. Tumor cells-expressed PIWIL1 may be a potential target for the development of novel HCC treatment.

## Introduction

Hepatocellular carcinoma (HCC) is one of the most malignant human cancers that has ranked as the third leading cause of cancer-related death all over the world.^1^ The morbidity and mortality of HCC are recently increasing in Western countries in contrast to the decreasing trend in Asia.^2^ Early diagnosis is difficult due to the late appearance of clinical manifestation and insignificant pathognomonic symptoms or signs,^3^ and treatment allocation is complex, and HCC often arises with other comorbidities.^4^ The prognosis of HCC patients remains very poor though a variety of treatments has been developed. This is not only because that HCC development is closely related to the presence of chronic liver diseases arisen from various risk factors,^2^ but also due to the complex nature of the liver as a significant metabolic and immunological organ that may foster a complicated hepatic microenvironment favoring tumor growth. Some previous studies have revealed that metabolites from cancer cells could shape the pro-tumoral microenvironment through regulating functional phenotypes of different immune populations.^5,6^ In particular, metabolic switch towards Warburg effects during the oncogenic changes of cancer cells was found to instigate the immunosuppressive network that favors cancer progression.^7^ Understanding the oncogenic activation of crosstalk between metabolic reprogramming and immune evasion in HCC may offer new opportunities in cancer therapy.

The PIWI proteins that belong to the Piwi clade of the Argonaute family are highly conserved and particularly expression during the animal germline development.^8^ Enlisting germline-specific Piwi-interacting RNAs (piRNAs), Piwi proteins function to suppress transposable elements and to safeguard the genomic integrity of germ cells.^9^ Accumulating evidence revealed that Piwi/piRNA machinery regulates the protein-coding in germ cells, and functions essentially for gametogenesis.^10^ This suggests the multiple functions of PIWI in gene regulation beyond silencing and was supported by the observation in round spermatids that the machinery can select a subset of ARE-containing mRNA for translation activation.^11^ PIWIL1, the most studied protein among this PIWI four-protein family, could ably regulate gene expression functioning in DNA damage response, cell cycle re-entry, apoptosis, cell proliferation, and tight junctions.^12–15^ Accumulating recent studies have put great attention on the fact that PIWIL1 was found overexpressed in human cancers of not only the reproduction system,^16,17^ where it physiologically functions, but also global types of tissues.^18–20^ The expression of PIWIL1 in HCC was reported elevated compared to that in adjacent hepatic tissues,^21^ which predicts the poor prognosis of patients after curative resection.^22^ The experimental test revealed that PIWIL1 knockdown reduced the migration and proliferation of HCC cells;^23^ however, how PIWIL1 regulates the global HCC progression remained unknown.

In this work, we tried to depict the role of PIWIL1 in regulating HCC progression. The PIWIL1-driven metabolic and immune reprogramming of HCC was investigated with their crosstalk being illustrated.

## Results

### PIWIL1 promotes in vitro proliferation and in vivo growth of HCC

**Fig. 1:**
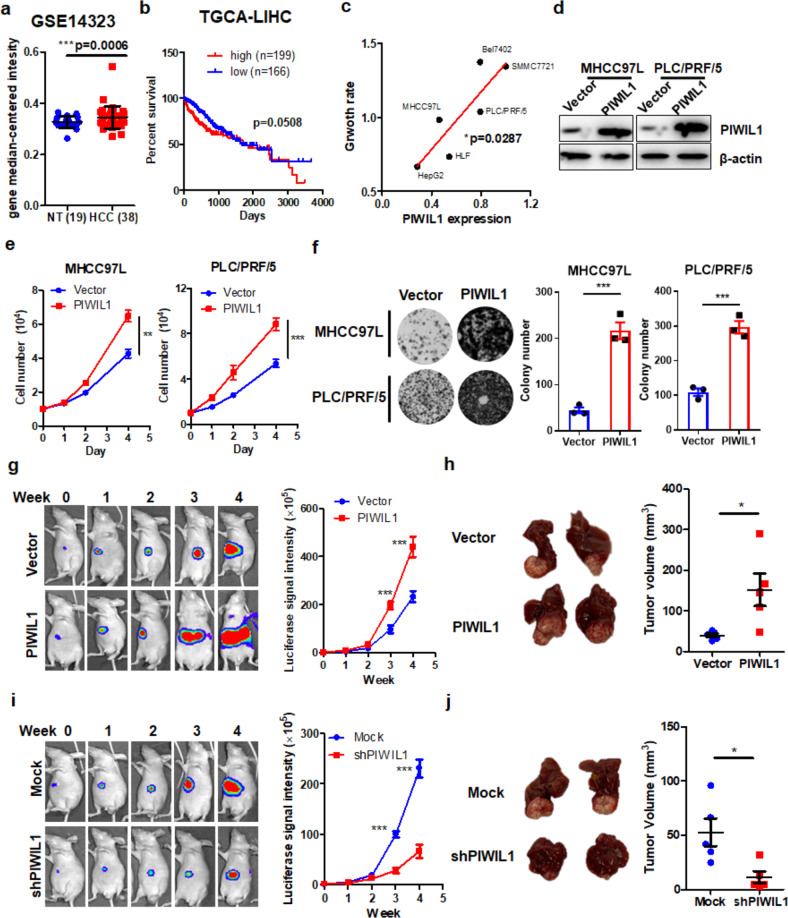
PIWIL1 promotes in vitro proliferation and in vivo growth of HCC. **a** Data were retrieved from GEO dataset. The expression of PIWIL1 was significantly higher in HCC tissue than normal liver in both GSE14323 dataset. The expression of PIWIL1 was significantly higher in HCC tissue than normal liver; **b** the overall survival data of HCC patients were retrieved from the TCGA database (*n* = 365). The expression of PIWIL1 has a weak correlation with the overall survival of HCC patients; **c** the mRNA expression of PIWIL1 in HepG2, MHCC97L, PLC/PRF/5, SMMC7721, Bel-7402, and HLF was measured by qRT-PCR. The cell proliferation rate was identified by calculating the value of the slope of the growth curve. The correlation was made and showed that the expression of PIWIL1 was positively correlated with the proliferation rate of the HCC cells; **d** Stable clones of PIWIL1-overexpressing MHCC97L and PLC/PRF/5 were established, and PIWIL1 overexpression was measured by immunoblotting; Overexpression of PIWIL1 could significantly promote the **e** proliferation and **f** colony formation of HCC cells. The orthotopic HCC model of mice was established with MHCC97L cells with or without PIWIL1 overexpression; **g** Overexpression of PIWIL1 could significantly intensify the luciferase signal intensity of MHCC97L cells, suggesting the weekly growth rate of orthotopic tumors was increased; **h** at the end of the study, the liver was dissected out. Tumors of HCC cells with PIWIL1 overexpression had a significantly larger size than those of wild-type cells (*n* = 5). Knockdown of PIWIL1 could reduce **i** the orthotopic growth and **j** size of HCC tumor in the liver of mice (*n* = 5). All experiments were performed in triplicate if without particular notice. *p < 0.05; **p < 0.01; ***p < 0.001

Several previous studies have depicted that PIWIL1 was overexpressed in HCC,^21,22^ while its role in mediating HCC progression remains unclear. To identify the clinical significance of PIWIL1, we first accessed the public database to retrieve the expression data of PIWIL1 in HCC datasets GSE14323. The expression of PIWIL1 was significantly upregulated in the tumors compared with normal hepatic tissue in the cohort of HCC patients (Fig. 1a). We then retrieved the survival data of HCC patients from the TGCA database. High expression of PIWIL1 has a weak correlation with HCC patients’ survival (Fig. 1b). To investigate whether overexpression of PIWIL1 facilitates the in vitro proliferation of HCC cells, we first screened different HCC cells and found there may be a correlation between the expression of PIWIL1 and their proliferation rates (Fig. 1c). Two HCC cell lines, MHCC97L and PLC/PRF/5, were selected for further study due to their intermediate levels of PIWIL1 expression as well as proliferation rate, which were suitable for both gain- and loss-of-function studies. We overexpressed PIWIL1 in both cells and measured the passage rate of PIWIL1-overexpressing HCC cells (Fig. 1d). Overexpression of PIWIL1 could significantly accelerate the proliferation of MHCC97L and PLC/PRF/5 cells (Fig. 1e). We then constructed an shRNA-PIWIL1-knockdown clone of MHCC97L and PLC/PRF/5 cells (Supplementary Fig. S1a) and observed a remarkably rate of proliferation in both cells when PIWIL1 was knockdown (Supplementary Fig. S1b). To assess the long-term growth, colony formation assay was performed. Overexpression of PIWIL1 could significantly induce an increasing ability of colony formation in both HCC cells (Fig. 1f), while the effect was opposite when PIWIL1 was knockdown (Supplementary Fig. S1c). To further examine if the PIWIL1-induced in vitro proliferation of HCC cells conferred to the in vivo tumor growth in the liver, we established murine models of both xenograft and orthotopic HCC. Overexpression of PIWIL1 could significantly induce tumor growth in HCC xenograft (Supplementary Fig. S1d, e), while PIWIL1 knockdown showed the opposite effect (Supplementary Fig. S1f, g). Using a luciferase reporter, we measured the growth rate of orthotopically implanted HCC tumors in the mice liver. Overexpression of PIWIL1 could potentially accelerate the weekly increase of luciferase signal intensity, suggesting the enlargement of implanted HCC tumors (Fig. 1g). At the end of the study, the liver was dissected out, in which significantly the more substantial size of PIWIL1-overexpressing HCC tumors was observed (Fig. 1h). On the contrary, the knockdown of PIWIL1 in MHCC97L resulted in a reduced growth rate (Fig. 1i) as well as the smaller size of end-point HCC tumors (Fig. 1j). Taken together, these observations confirmed that PIWIL1 could facilitate in vitro cell proliferation and in vivo tumor growth in HCC.

### PIWIL1 facilitates fatty acid metabolism to support the rapid progress of HCC

**Fig. 2:**
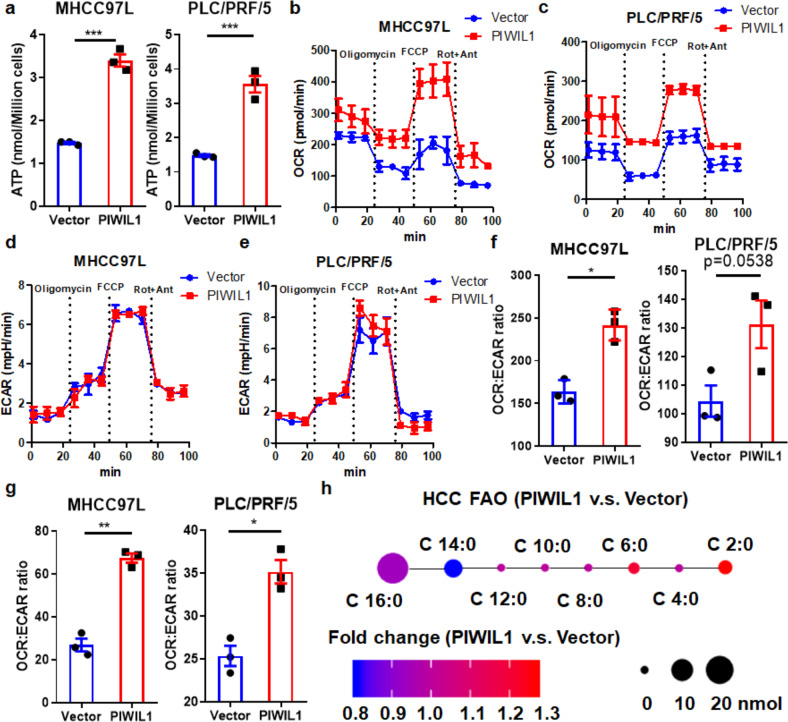
PIWIL1 modulates fatty acid oxidation in HCC cells. **a** Intracellular ATP of PIWIL1-overexpressing HCC cells was significantly higher than that of wild-type cells; Overexpression of PIWIL1 could significantly increase the oxygen consumption rate of HCC cells, as indicated by OCR assay (**b**, **c**), while its effect on ECAR of HCC cells was not significance (**d**, **e**). Overexpression of PIWIL1 led to the higher value of OCR/ECAR ratio at basal (**f**) and maximal (**g**) respiration. **h** The composition of fatty acid metabolites of wild type and PIWIL1-overexpressing HCC tumors was analyzed by GC-MS, which indicated an increase of C16 intermediates (*n* = 5). All experiments were performed in triplicate if without particular notice. *p < 0.05; **p < 0.01; ***p < 0.001

Cancer cells require a large amount of energy in a short period to proliferate rapidly.^24^ To suppose this, cancer cells were usually accelerating glucose uptake and utilizing anaerobic glycolysis to generate more energy in supporting the demands of rapid tumor progression.^25^ Interestingly, overexpression of PIWIL1 in HCC cells resulted in a significant increase of intracellular ATP, suggesting that PIWIL1 may promote energy production to support rapid cell proliferation (Fig. 2a). To figure out whether the induction of HCC progression by PIWIL1 involves increasing energy metabolism, we measured if PIWIL1 could manipulate the metabolic switch in HCC cells. Surprisingly, we did not observe any significant increase in glucose uptake of HCC cells by PIWIL1 overexpression (Supplementary Fig. S2a). The extracellular and intracellular level of lactate, a glycolytic metabolite, was not significantly changed (Supplementary Fig. S2b, c). Furthermore, no significant changes in expression of genes related to glycolysis, such as *Hk2*, *Pkm2*, and *Ldha*, were observed, which collectively indicated that PIWIL1 might not alter the glycolytic pathway to accelerate energy metabolism (Supplementary Fig. S2d). To further measure the metabolic profile of PIWIL1-overexpressing HCC cells, we performed the extracellular acidification rate (ECAR) and oxygen consumption rate (OCR) assays using the Seahorse XF machine. PIWIL1 overexpression resulted in a remarkable increase in oxygen consumption at both basal and maximal rate of metabolism (Fig. 2b, c), while the increase in ECAR was not as significant (Fig. 2d, e), suggesting that PIWIL1-overexpressing HCC cells may preferably utilize oxidative phosphorylation (OXPHOS) to generate ATP. This was further supported by the observation that mitochondrial reactive oxygen species (ROS) was markedly elevated when PIWIL1 was overexpressed in HCC cells (Supplementary Fig. S2e). Furthermore, PIWIL1 overexpression significantly increased the basal and maximal respiratory capacity (SRC) of MHCC cells. Increased OCR/ECAR in PIWIL1-overexpressing PLC/PRF/5 cells, but the statistical significance at basal respiratory capacity was very margin (*p* = 0.0538) (Fig. 2f, g). As mitochondrial fatty acid β-oxidation (FAO) may significantly contribute to the SRC in OXPHOS,^26^ we hypothesized that PIWIL1 might accelerate FAO in HCC cells. Consumption of intracellular cellular free fatty acid was increased in PIWIL1-overexpressing HCC cells (Supplementary Fig. S2f). Metabolic profiling of fatty acid derived from PIWIL1-overexpressing tumors suggested an increased C16 intermediate compared with that from wild type tumors (Fig. 2h).^27^ FAO assay using oleate as a substrate further confirmed that PIWIL1 could promote fatty acid metabolism in HCC cells (Supplementary Fig. S2g). These findings suggested that promote fatty acid metabolism in accelerating energy production for the demands of rapid tumor growth.

### PIWIL1-induced fatty acid metabolism is associated with its regulation on immune cells

**Fig. 3:**
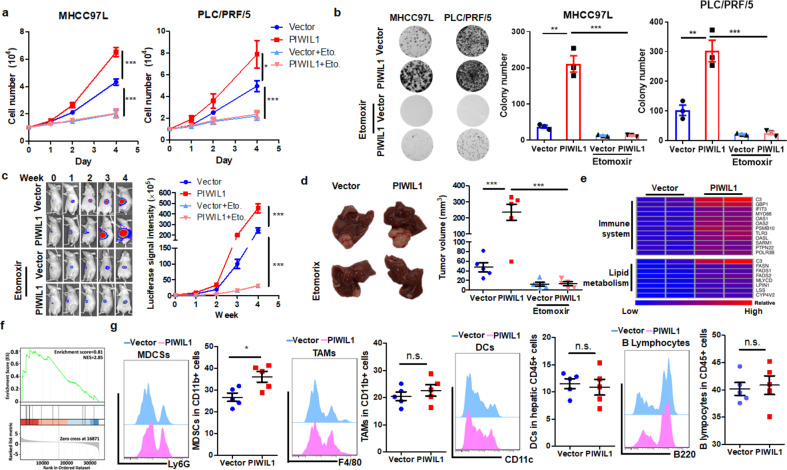
PIWIL1-regulated FAO is associated with the regulation of the immune microenvironment of HCC. Wild type and PIWIL1-overexpressing HCC cells were tested in the presence of 100 μM etomoxir. The **a** proliferation and **b** colony formation ability of PIWIL1-overexpressing HCC were significantly suppressed by etomoxir treatment; Orthotopic models of wild type and PIWIL1-overexpressing HCC were established, and mice were treated with etomoxir (50 mg/kg) every other day by intraperitoneal injection. The growth rate (**c**) and tumor size (**d**) of both vector- and PIWIL1-overexpressing HCC were significantly reduced in the presence of etomoxir (*n* = 5); **e** Vector-expressing and PIWIL1-overexpressing PLC/PRF/5 cells collected and total RNA was extracted. RNA sequencing was performed to analyze the difference in gene expression. Heatmap of some genes with significant expression was generated. **f** GSEA analysis of gene sets associated with the immune system process was performed. **g** The whole liver of mice with orthotopically implanted HCC was digested. The population of TAMs, DCs, B lymphocytes, and NK cells were analyzed by flow cytometry using a particular cell surface marker (*n* = 5). All experiments were performed in triplicate if without particular notice. *p < 0.05; **p < 0.01; ***p < 0.001

To further understand the role of fatty acid oxidation as a facilitator of energy support in PIWIL1-overexpressing cells, we used Etomoxir, an inhibitor of the carnitine transporter CPT1,^28^ to block the FAO process in PIWIL1-overexpressing HCC cells. The presence of etomoxir could significantly reduce the fatty acid oxidation rate (Supplementary Fig. S3a) as well as the production of ATP (Supplementary Fig. S3b) in PIWIL1-overexpressing HCC cells. Cell proliferation assay suggested that the presence of etomoxir could significantly suppress the PIWIL1-induced cell growth (Fig. 3a), and colony formation assay supported the same claim (Fig. 3b). Besides, the treatment of etomoxir could significantly reduce the xenograft growth rate of PIWIL1-overexpressing HCC in mice (Supplementary Fig. S3c), as well as the size of the tumor (Supplementary Fig. S3d), although treating etomoxir may also restrict the tumor growth in wild type HCC. This effect of etomoxir was reproducibly observed in mice with orthotopically implanted HCC tumors overexpressing PIWIL1, in which the weekly growth rate of both wild type and PIWIL1-overexpressing tumors was reduced, while PIWIL1-overexpressing HCC tumor showed a greater decrease in growth than its wild type counterpart (Fig. 3c). The size of hepatic tumors showed a consistent trend of changes (Fig. 3d). These in vitro and in vivo observations confirmed that PIWIL1-induced FAO in HCC cells contributes to, as least in part, the progression of HCC.

We then performed RNA-seq to map the possible downstream network of fatty acid metabolism in PIWIL1-overexpressing cells. Vector-expressing or PIWIL1-overexpressing PLC/PRF/5 cells were collected for the analysis. It was observed that overexpression of PIWIL1 could induce a series of changes in gene expression (Fig. 3e). The expression of selected genes was verified by qPCR to validate the RNA-seq results (Supplementary Fig. S3e, f). We shortlisted genes with significant changes in response to PIWIL1 overexpression. Genes upregulated or downregulated were then put into Gene Ontology (GO) analysis to enrich possible biological processes (Supplementary Fig. S3g, h), and it was interesting to find that PIWIL1-induced gene expression may be ably regulating the immune system and fatty acid metabolism of HCC. To further understand the link between PIWIL1 overexpression-induced FAO and HCC cell proliferation, we reviewed the RNA sequencing results and identified MLYCD as one of the FAO-related genes upregulated by PIWIL1 overexpression. Human MLYCD encodes Malonyl-CoA decarboxylase that catalyzes the conversion of malonyl-CoA into acetyl-CoA and carbon dioxide and has been proven by the previous study to promote FAO in cancer cells.^29^ We, therefore, hypothesize that MLYCD may be the downstream factor involving in PIWIL1-mediated FAO-associated HCC proliferation. It was noted that knockdown of MLYCD significantly attenuated the PIWIL1-induced HCC cell proliferation and colony formation (Supplementary Fig. S3i–k). This in vitro observation combined with other observations strongly supports the role of FAO in mediating PIWIL1-induced HCC growth. On the other hand, GSEA indicated that gene sets associated with the immune system process have a strong positive correlation with PIWIL1 overexpression (Fig. 3f). Population analysis using flow cytometry showed a significant increase of CD11b + Ly6G+ myeloid-derived suppressor cells (MDSCs) in the HCC-bearing livers of athymic nude mice. In contrast, the hepatic populations of CD11b + F4/80+ tumor-associated macrophages (TAMs), CD11b + CD11c + dendritic cells (DCs), and B220 + B cells remained insignificantly changed (Fig. 3g). These findings connect the PIWIL1-induced fatty acid metabolism with its regulation of the immune cell process in HCC.

### MDSCs population is responsible for PIWIL1-induced immunosuppression in HCC

**Fig. 4:**
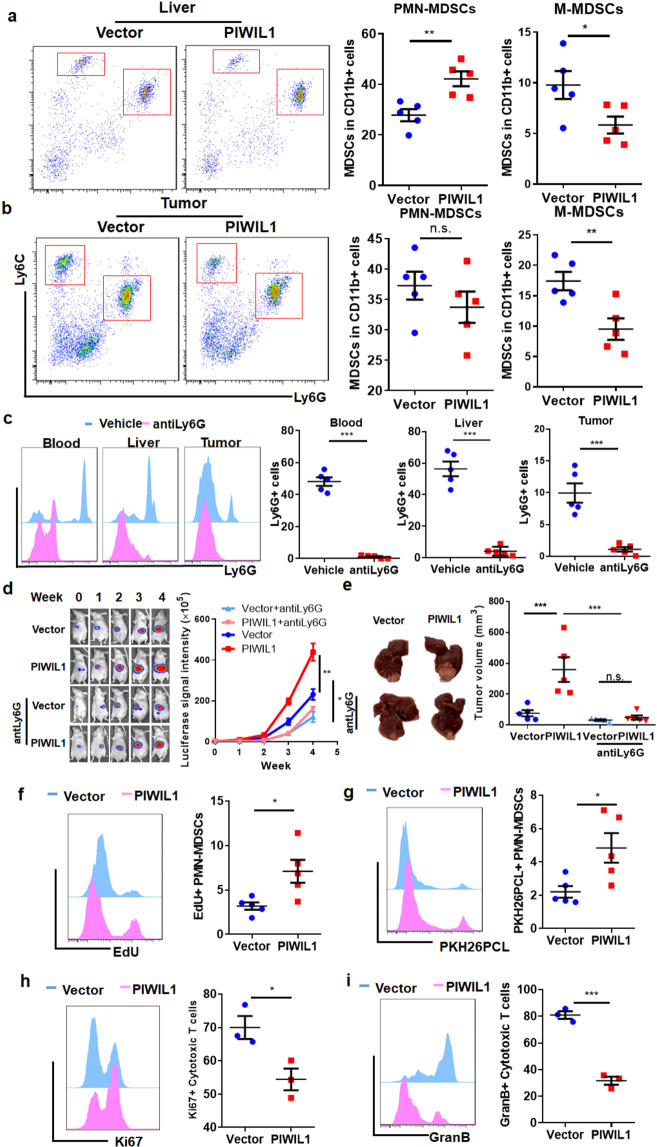
PIWIL1 overexpression-induced MDSCs in the tumor microenvironment of HCC. The tumor and surrounding hepatic tissues (within 5 mm from the tumor) of orthotopic HCC mice were collected. The population of analysis of MDSCs was analyzed by flow cytometry; PIWIL1 overexpression significantly accumulated the PMN-MDSCs in the surrounding hepatic tissues (**a**), while the M-MDSCs population was suppressed (**b**); We then used anti-Ly6G to deplete the PMN-MDSCs population. Intraperitoneal injection of anti-Ly6G antibody (200 mg/kg, every 4 days) successfully suppressed the PMN-MDSCs population (**c**) and attenuated the PIWIL1-induced HCC growth (**d**) and tumor size (**e**); **f** EdU was intraperitoneally injected into the mice (10 mg/kg). Five hours after injection, mice were sacrificed and the EdU-incorporated PMN-MDSCs were analyzed by flow cytometry. Increased EdU incorporation was observed in PMN-MDSCs of PIWIL1-overexpressing orthotopic tumor; **g** BMDMs were isolated and stained by PKH26PCL. The PKH26PCL-positive BMDMs were then intraperitoneally injected (1 × 10^7^ cells/mice) 48 h before sacrifice. The infiltration of PKH26PCL-positive cells was significantly increased in the hepatic tissues surrounding PIWIL1-overexpressing HCC; the PMN-MDSCs in the hepatic tissues surrounding wild type or PIWIL1-overexpressing HCC were sorted and co-cultured with simulated CD8 + cytotoxic T cells. PMN-MDSCs from PIWIL1-overexpressing HCC had a more potent ability in inhibiting the expression **h** Ki67, a marker of cell proliferation, and **i** Granzyme B (GranB), a marker of activation of co-cultured stimulated CD8 + cytotoxic T cells. All experiments were performed in triplicate. *p < 0.05; **p < 0.01; ***p < 0.001

As a cancer hallmark discovered in recent years, the immunosuppressive microenvironment surrounding the tumor cells was composed of a series of immune and non-immune components.^30^ Tumor cells tend to foster an immunosuppressive microenvironment to facilitate its progression and metastasis, in which several types of immune cells such as TAMs and MDSCs, play a crucial role.^31^ As an increase in the MDSCs population was observed, we further depicted the detailed profile of MDSCs in PIWIL1-overexpressing HCC. Sub-population of granulocytic MDSCs (PMN-MDSCs, CD11b + Ly6G + Ly6C-) and monocytic MDSCs (M-MDSCs, CD11b + Ly6G-Ly6C^hi^) were analyzed. It was observed that PIWIL1-overexpressing orthotopic tumor significantly attracted PMN-MDSCs from the circulation; however, PMN-MDSCs harbored at the liver tissues surrounding the HCC tumor without further infiltrating into the tumor tissue. On the contrary, reduced infiltration of M-MDSCs in the liver and tumor tissues were observed in PIWIL1-overexpressing HCC tumors (Fig. 4a, b). To investigate if the infiltration of PMN-MDSCs in the surrounding hepatic tissues contributes to the tumor-promoting effect of PIWIL1 in HCC, we depleted this population using a specific anti-Ly6G antibody. Vast depletion of PMN-MDSCs was observed in the blood, liver, and tumor tissues after antibody injection (Fig. 4c), and the PIWIL1-induced increased growth of HCC tumors was found partially attenuated (Fig. 4d), and the end-point tumor size was suppressed (Fig. 4e), suggesting a revoking immune system upon MDSCs depletion may counteract, at least partially, the tumor-promoting effect of PIWIL1 in HCC. To further confirm that PIWIL1 promotes HCC growth through fostering immunosuppressive MDSCs, HCC-bearing mice were pre-treated with anti-Ly6G antibody and randomized into two groups. The mice were then received adoptive transfer of 2 × 10^6^ BMDMs cultured with HCC supernatant from either vector- or PIWIL1-overexpressing MHCC97L cells every 4 days via intraperitoneal injections for 4 weeks (Supplementary Fig. S4a). The supernatant of PIWIL1-overexpressing HCC cells was proven in the present study to induce a higher level of MDSCs than vector-expressing HCC cells in in vitro culture of isolated BMDMs. We observed that orthotopic tumor implanted mice receiving adoptive transfer of MDSCs cultured with PIWIL1-overexpressing HCC cell supernatant exhibited significantly accelerated growth and larger tumor size than those with counterparts receiving MDSCs cultured with vector-expressing HCC cell supernatant (Fig. S4b, c). This observation in the adoptive transfer experiment combined with MDSCs depletion by anti-Ly6G antibody supported that MDSCs are involved in mediating PIWIL1-induced HCC growth in vivo. To identify if PIWIL1-overexpressing HCC cells directly lead to the accumulation of PMN-MDSCs in the surrounding hepatic tissues, we cultured bone marrow-derived monocytes (BMDMs) with conditioned medium derived from PIWIL1-overexpressing HCC cells. A significant increase of CD11b + Ly6G + Ly6C- population was observed in BMDMs cultured with conditioned medium from PIWIL1-overexpressing HCC cells (Supplementary Fig. S4d), while the population of CD11b + Ly6G-Ly6C^hi^ cells was reduced (Supplementary Fig. S4e). This effect seemed to have time manner, as the increase of CD11b + Ly6G + Ly6C- cells by conditioned medium derived from PIWIL1-overexpressing HCC cells enlarged after 4-day culture (Supplementary Fig. S4f). Expression of MDSCs-related genes, such as *Arg1*, *Nos2*, and *Il10*, was induced (Supplementary Fig. S4g). Also, MDSCs induced by conditioned medium derived from PIWIL1-overexpressing HCC cells exhibited a higher proliferation rate as detected by BrdU assay (Supplementary Fig. S4h), as well as increased migration ability (Supplementary Fig. S4i), which may resemble the mechanism of PMN-MDSCs assembly in the hepatic tissues surrounding the HCC tumors. To investigate if it is the case in vivo, we detected a local proliferation of PMN-MDSCs with EdU incorporation assay. More EdU was incorporated into the DNA of hepatic PMN-MDSCs cells surrounding PIWIL1-overexpressing HCC tumors, suggesting that local PMN-MDSCs proliferated more rapidly in response to PIWIL1 overexpression in tumors (Fig. 4f). In the meaning time, PKH26PCL-stained monocytes were intraperitoneally injected into the mice orthotopically bearing wild type and PIWIL1-overexpressing HCC tumors to trace the migration of MDSCs. Significant accumulation of PKH26PCL-positive PMN-MDSCs in the hepatic tissue surrounding PIWIL1-overexpressing HCC tumors (Fig. 4g). These observations suggest that the proliferation and infiltration of PMN-MDSCs in the surrounding hepatic tissues may contribute to the immunosuppressive microenvironment of PIWIL1-overexpressing HCC.

### PIWIL1-induced MDSC immunosuppression was associated with P38 MAPK-mediated IL10 production

**Fig. 5:**
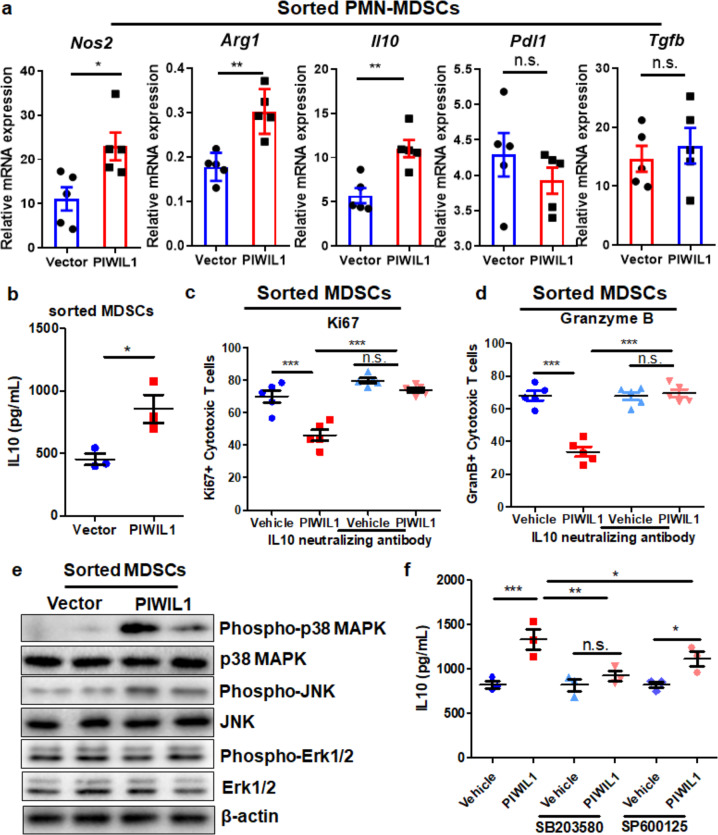
MDSCs of PIWIL-overexpressing HCC suppresses T-cell proliferation and activation through IL10-dependent manner. The PMN-MDSCs in the hepatic tissues surrounding wild type or PIWIL1-overexpressing HCC were sorted and cultured. Significantly higher expression of PMN-MDSCs genes (**a**) and IL10 production (**b**) were observed in PMN-MDSCs from PIWIL1-overexpressing HCC; The PMN-MDSCs in the hepatic tissues surrounding wild type or PIWIL1-overexpressing HCC were sorted and co-cultured with simulated CD8 + cytotoxic T cells in the presence of IL10 neutralizing antibody. IL10 neutralizing antibody could potentially recover the **c** Ki67 and **d** Granzyme B expression in these T cells; **e** Protein was extracted from sorted PMN-MDSCs, and the phosphorylation of p38 MAPK and JNK were found induced in sorted PMN-MDSCs from MDSCs induced by conditioned medium from PIWIL1-overexpressing HCC cells; BMDMs was incubated with conditioned medium from PIWIL1-overexpressing HCC cells following pre-incubation of p38 MAPK inhibitor SB203580 (10 μM) or JNK inhibitor SP600125 (10 μM) for 60 min. The protein secretion of IL10 was significantly suppressed by SB203580 or SP600125 (**f**). All experiments were performed in triplicate. *p < 0.05; **p < 0.01; ***p < 0.001

The immunosuppressive activity of MDSCs in wild type and PIWIL1-overexpressing HCC tumors was then confirmed by T-cell suppression assay. MDSCs treated with conditioned medium derived from wild type and PIWIL1-overexpressing HCC cells were co-cultured with sorted CD3 + CD8 + cytotoxic T cells stimulated with anti-CD3e and anti-CD28 antibodies for 72 h. Significantly reduced Ki67 and Granzyme B expression in the cytotoxic T cells after exposure to MDSCs treated with conditioned medium derived from PIWIL1-overexpressing HCC cells were observed (Supplementary Fig. S4j, k), suggesting T-cell proliferation and activation were suppressed. We then sorted intrahepatic PMN-MDSCs surrounding the wild type and PIWIL1-overexpressing HCC tumors, and the observed a more potent immunosuppressive activity of PMN-MDSCs from PIWIL1-overexpressing tumors to T-cell proliferation and activation than those from wild type tumors (Fig. 4h, i). MDSCs render immunosuppressive effects in the tumor microenvironment through various mechanisms. To explore which mechanism may play a primary role in the immunosuppression of MDSCs in PIWIL1-overexpressing HCC tumors, we sorted PMN-MDSCs from the surrounding hepatic tissues of wild type and PIWIL1-overexpressing HCC and measured the expression of several primary effectors, such as *Il10*, *Arg1*, *Nos2*, *Tgfb*, and *Pdl1*. MDSCs-secreting IL10 can foster an immunosuppressive microenvironment by targeting both acquired and innate immune cells, or directly prevent cell death of tumor cells;^32,33^ Arginase and iNOS in MDSCs competitively consume arginine in the microenvironment with T cells and prevented T-cell activation and expansion;^34^ Expression of PDL1 in MDSCs interacted with PD1 on cytotoxic T cells to cause its exhaustion;^35^ TGF-β produced by MDSCs has an anti-inflammatory effect and can switch towards immunosuppressive microenvironment to support tumor growth.^36^ Significant induction of gene expression of *Il10*, *Arg1*, and *Nos2* was observed in PMN-MDSCs from PIWIL1-overexpressing HCC, while *Tgfb* and *Pdl1* remained unchanged (Fig. 5a). Significant induction of corresponding protein expression of IL10, Arginase-1, and iNOS was also observed (Fig. 5b and Supplementary Fig. S5a). To identify the primary pathway involved in the immunosuppressive activity of MDSCs induced by PIWIL1-overexpressing tumors, we first supplemented the Arginase-1 substrate l-arginine, or the iNOS inhibitor aminoguanidine, to the co-culture of stimulated T cells and MDSCs treated with conditioned medium derived from wild type and PIWIL1-overexpressing HCC cells. Unexpectedly, the re-supplementation of L-arginine (Supplementary Fig. S5b, c), or presence of aminoguanidine (Supplementary Fig. S5d, e), had minimal effect on the proliferation and activation of co-cultured T cells. The addition of neutralizing antibodies against IL10 could significantly improve the proliferation and activation of stimulated cytotoxic T cells co-cultured with MDSCs treated by conditioned medium from PIWIL1-overexpressing HCC cells (Supplementary Fig. S5f, g), as well as stimulated cytotoxic T cells co-cultured with sorted MDSCs from PIWIL1-overexpressing HCC (Fig. 5c, d).

We checked the level of IL10 in the tumor and hepatic tissues surrounding the tumors. Although both tumors and the surrounding hepatic tumor was infiltrated with a higher level of IL10 than normal liver tissues, the difference of IL10 level in tumor and its surrounding hepatic tissues is not significant (Supplementary Fig. S5h). Previous studies have shown that the anti-inflammatory IL10 was overexpressed in both intratumoral and peritumoral hepatic tissues of HCC patients,^37^ and has delivered multiple tumor-promoting functions in both areas of the liver. Although IL10 is majorly secreted by cells accumulated at the hepatic tissues surrounding liver tumors,^37,38^ IL10 secretion into the tumors can induce dysregulation of the immune population including intratumoral T cells.^39,40^ At the same time, a high level of IL10 in the hepatic tissues surrounding HCC tumors could regulate the peritumoral immune populations to deliver a significant immunosuppressive effect.^37^ Besides, IL10 may directly foster the proliferation of tumor cells and lead to cancer progression.^33^ In our study, in addition to the observation that MDSCs-secrete IL10 can regulate immune functions, the in vitro co-culture study showed that HCC cells co-cultured with sorted wild type and PIWIL1-overexpressing MDSCs exhibited increased proliferation rate, which can be blocked by the presence of neutralizing antibody of IL10 (Supplementary Fig. S5i).

The MAPKs signaling has been found to primarily mediate the immunosuppressive functions through both regulating Arginase/iNOS balance and cytokine production.^41^ As in our study, we observed MDSCs sorted from PIWIL1-overexpressing HCC tumors exhibited higher levels of both Arginase/iNOS and IL10 cytokine, we focused on the regulation of MAPKs signaling. Three members of MAPKs, including p38 MAPK, Erk1/2, and JNK was measured. Significant activation of p38 MAPK and JNK was observed in sorted PMN-MDSCs treated with conditioned medium from PIWIL1-overexpressing HCC cells, Erk1/2 activity remained unchanged (Fig. 5e). To further understand the role of p38 MAPK and JNK in mediating interleukin (IL)-10 production, we used p38 MAPK inhibitor and JNK inhibitor, SB203580, and SP600125, respectively, to block the signal activation. Pre-treatment of both 10 μM SB20350 and SP600125 for 60 min can attenuate the increased production of IL10 in MDSCs cultured with conditioned medium from PIWIL1-overexpressing HCC cells (Fig. 5f). SB203580 and SP600125 can target both p38 MAPK and JNK with differential potencies. While P38 MAPK is more sensitive to SB203580, JNK is more responsive to SP600125.^42,43^ In our study, we found that SB203580 exhibited stronger inhibition than SP600125 on the increased production of IL10 in MDSCs cultured with conditioned medium from PIWIL1-overexpressing HCC cells. Although this result cannot directly conclude that p38 MAPK but not JNK was responsible for the IL10 production by MDSCs from PIWIL1-overexpressing HCC tumors, as a previous study found that both p38 MAPK and JNK inhibition can lead to downregulation of IL10,^44^ the observation that SB203580 has more potent inhibition than SP600125 on the IL10 production may suggest that p38 MAPK plays a more important role in mediating IL10 production by MDSCs of PIWIL1-overexpressing HCC tumors.

### PIWIL1-induced complement C3 fosters MDSCs in the tumor microenvironment of HCC

**Fig. 6:**
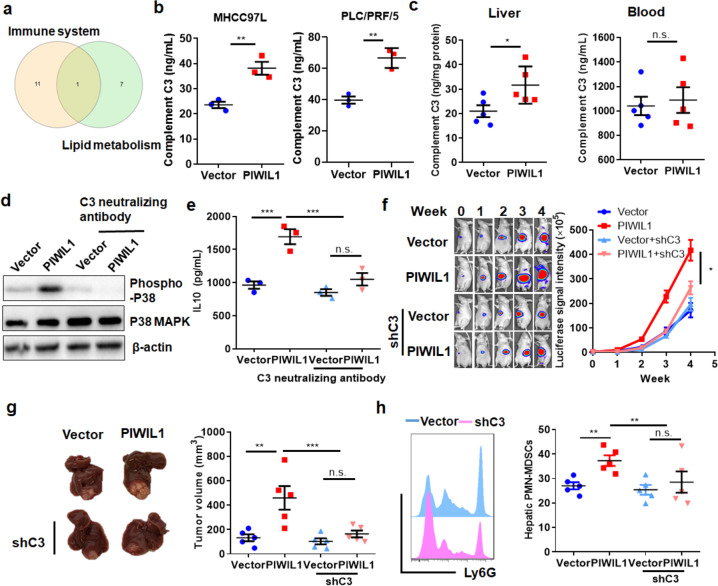
PIWIL1-induced Complement C3 expression in HCC cells regulated the immunosuppressive activity of HCC. **a** Gene lists in two enriched clusters, immune system regulation, and lipid metabolism regulation were overlapped. Complement C3 was the only common gene in both clusters; **b** the secretion of complement C3 was measured in wild type and PIWIL1-overexpressing HCC cells, which showed that PIWIL1 overexpression could remarkably induce C3 secretion in HCC cells; **c** Complement C3 level was also then measured in the circulating blood and surrounding hepatic tissues of mice with wild type or PIWIL1-overexpressing HCC. Increased complement C3 level in surrounding hepatic tissues but not circulating blood of mice with PIWIL1-overexpressing HCC was observed. The BMDMs were isolated using ficoll method, and cultured with 50% conditioned medium from PIWIL1-overexpressing HCC cells for 4 days in the presence of neutralizing antibodies against human complement C3 protein. Blockade of Complement C3 significantly reduced **d** the activation of p38 MAPK and **e** secretion of IL10; A stable C3 knockdown clone of PIWIL1-overexpressing MHCC97L cells was constructed. The orthotopic HCC model was established with PIWIL1-overexpressing tumor cells with or without C3 knockdown. Knockdown of C3 in PIWIL1-overexpressing HCC cells could significantly attenuate **f** growth rate, **g** tumor size of PIWIL1-overexpressing HCC in mice, and **h** potently suppress the infiltration and accumulation of PMN-MDSCs at the hepatic tissue surrounding PIWIL1-overexpressing HCC tumors (*n* = 5). All experiments were performed in triplicate if without particular notice. *p < 0.05; **p < 0.01; ***p < 0.001

To further identify the connection between the cellular actions of PIWIL1 in HCC cells with its induction on immunosuppressive properties of MDSCs, we re-visited the RNA-seq data. Overlap of gene sets involved in the biological processes of the immune system process and lipid metabolism process suggested that Complement C3 was the common protein across these two critical events of PIWIL1 overexpression in HCC cells (Fig. 6a). mRNA expression of *C3* was induced in HCC cells overexpressing PIWIL1 and was suppressed in cells with PIWIL1 knockdown (Supplementary Fig. S6a). Consistently, the secretion of complement C3 protein from HCC cells was induced by PIWIL1 overexpression (Fig. 6b). Moreover, we observed a potent elevated C3 level in the hepatic tissues surrounding PIWIL1-overexpressing HCC tumors mice with insignificant changes at its circulating level (Fig. 6c). While a few studies showed that complement C3 can regulate fatty acid metabolism,^45^ control of cellular FAO on complement C3 was never reported. This may be due to the complicated processes of FAO and multiple side products being produced, which could regulate C3 expression. In our study, we found that FAO induced by PIWIL1 overexpression can significantly increase the mitochondrial ROS production that led to oxidative stress. It was previously showed that oxidative stress in the cells is one of the mechanisms of Complement C3 activation.^46^ In this case, we used a mitochondrial ROS scavenger, catalase, to relieve oxidative stress. The presence of catalase in PIWIL1-overexpressing HCC cells could significantly abolish Complement C3 expression (Supplementary Fig. S6b), which indicated that FAO-mediated ROS production is at least partially, if not all, involved as a potential mechanism of Complement C3 activation in PIWIL1-overexpressing HCC cells.

To understand if the induction of Complement C3 in PIWIL1-overexpressing HCC cells mediates the enhanced immunosuppressive activity of MDSCs, we used a monoclonal antibody to neutralize the Complement C3 in the conditioned medium derived from PIWIL1-overexpressing HCC cells. Neutralization of C3 attenuated the induction of p38 MAPK signaling activation (Fig. 6d), as well as IL10 production (Fig. 6e) in MDSCs, treated with the conditioned medium, indicating that C3 may play a critical role in enhancing the immunosuppressive activity of MDSCs in PIWIL1-overexpressing HCC. Supplementation of C3 recombinant protein in the conditioned medium of wild type HCC cells evoked its ability in inducing the population of CD11b + Ly6G + Ly6C- MDSCs (Supplementary Fig. S6c), with activation of its intracellular P38 MAPK signaling (Supplementary Fig. S6d) and IL10 production (Supplementary Fig. S6e). To examine the role of C3 in vivo, we knocked down the C3 expression in PIWIL1-overexpressing HCC cells by shRNA to construct a stable clone of PIWIL1-overexpressing MHCC97 cells with C3 knockdown (Supplementary Fig. S6f). Knockdown of C3 in PIWIL1-overexpressing cells, at least in part, delayed the growth of orthotopic HCC tumors in the mice liver (Fig. 6f, g). The secretion of C3 into the surrounding hepatic tissues was reduced (Supplementary Fig. S6g), as expected, and this was accompanied by the reversal of PMN-MDSCs infiltration into these tissues (Fig. 6h). These observations indicated that complement C3 could be responsible for the PIWIL1-induced MDSCs accumulation in the tumor microenvironment of HCC.

## Discussion

**Fig. 7:**
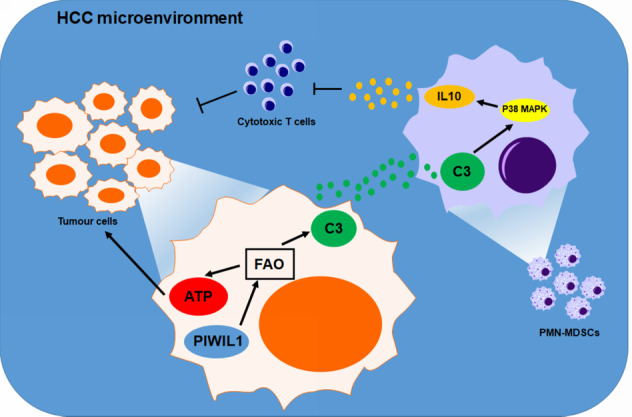
Schematic regulation mechanism of PIWIL1 in HCC

In this study, we observed PIWIL1-mediated crosstalk of metabolic and immune systems of HCC with complement C3 as a scaffold protein that plays a role (Fig. 7). Unexpectedly we observed an accelerated fatty acid metabolism instead of anaerobic glycolysis in fueling HCC progression upon PIWIL1 overexpression Evidence of dysregulation of FAO as an energy source in cancer cells was limited. However, overexpression of FAO-related proteins was frequently reported.^47^ HCC cells may accelerate FAO to maintain the energy homeostasis to counteract stress-induced cancer cell death.^48^ Some specific cell types in the tumor stroma of HCC, such as tumor-initiating cells^49^ and TAMs,^27^ were found to utilize FAO to generate more ATP for the initiation and progression of HCC. Our findings here showed that PIWIL1 drives FAO as a source of ATP generation in differentiated HCC cells, which was responsible for PIWIL1-associated tumor progression. Mechanisms underlying PIWIL1-induced FAO remained to be inspected in detail. However, a previous study observed that β-catenin-activated HCC might process FAO for energy production through a PPARɤ-dependent mechanism.^50^ Our RNA-seq analysis suggested that PIWIL1 could significantly up-regulate the expression Lipin-1, a coactivator of PPARɤ that is required for the transcriptional activity of the protein.^51^ Lipin-1 deficiency was shown to result in impairment of FAO in human subjects.^52^ Although direct induction of PPARɤ expression as a transcription factor of FAO enzymes was not observed, up-regulation of its coactivator by PIWIL1 may promote the activity of PPARɤ to facilitate the transcription of FAO-associated genes.

We observed that the PIWIL1-induced metabolic switch towards FAO in HCC might create an immunosuppressive microenvironment that facilitated cancer progression. Suppression of FAO in the tumor microenvironment resulted in inhibition of MDSCs and activation of CD8 + cytotoxic T cells that delayed tumor growth.^53^ These findings cannot rule out the possibility of the antitumor effect of FAO inhibition within MDSCs, as several studies have suggested that the intracellular FAO in MDSCs could directly modulate its immunosuppressive activity in cancer therapies.^54^ Our findings in the current study suggest that modulation on the FAO of tumor cells may also play a role in regulating the immunosuppressive microenvironment. This was in accordance with some previous studies, for example, Li et al. revealed that in retinoblastoma FAO in tumor cells facilitated secretion of CCL2 that globally attracted a series of the immunosuppressive population including MDSCs, TAMs, and Tregs.^55^ Interestingly, instead of observing a mast infiltration of MDSCs into PIWIL1-overexpressing HCC tumors, we found most of the immunosuppressive MDSCs harbored accumulatively at the hepatic tissues surrounding the tumors. Although we still have no idea why MDSCs preferably stayed at the tissues neighboring HCC tumors, this accumulation seemed not to affect the formation of an immunosuppressive microenvironment. Indeed, several previous studies have suggested the immunosuppressive population of immune cells, such as Treg and M2-like TAMs, did not directly infiltrate into the HCC tumors but harbored at surrounding hepatic tissues of mice and human, which sufficiently developed a microenvironment favoring HCC growth.^56,57^ We observed an increased population of TAMs in PIWIL1-overexpressing HCC tumors (data not shown). Considering the heterogeneity of the cells, MDSCs may rapidly differentiate into TAMs when infiltrating into the tumor tissues, resulting in the reduction of MDSCs in number in the tumor areas.^34^ Another possible interpretation is that MDSCs may be attracted by several types of non-parenchymal populations, such as Kuffer cells and hepatic stellate cells, and harbored at the surrounding non-tumor tissues to facilitate an immunosuppressive microenvironment.^58,59^

We found that C3 secretion was responsible for delivering the immunosuppressive signal from PIWIL1-overexpressing HCC to its microenvironments. As an essential component of innate immunity, complement activation mediates the cell-cell interaction and regulates the extravascular tissues in the tumor microenvironment, and therefore enhances tumor growth and metastasis.^60^ Supplementation of complement C3 and C5 may facilitate the tumor growth, and in particular, it was previously reported that the pro-tumoral effect of complement C5 is associated with its regulation on the immunosuppressive activity of MDSCs.^61^ It was recently observed that in HCC, hepatic stellate cells might produce C3 to regulate the MDSCs to foster a pro-tumoral microenvironment.^62,63^ Another study reported that depletion of C3 from tumor cells could facilitate tumor immunotherapy, suggesting the role of C3 in suppressing antitumor immunity.^64^ This was in accordance with our observation that PIWIL1 overexpression in tumor cells could lead to more C3 secretion to modulate the environmental MDSCs. Besides, we found that PIWIL1-induced C3 secretion could activate P38 MAPK-mediated IL10 production. IL10 was considered as the significant inhibitory factors of antitumor immunity in cancer upon complement activation.^65^ P38 MAPK was found responsible for C3-induced IL10 production in MDSCs. This was supported by a previous observation that the cleavage product of C3 could bind to the C3a receptor in BMDMs that amplifies the downstream p38 MAPK signaling.^66^ Activation of p38 MAPK mediates both pro-inflammatory and anti-inflammatory responses; however, the mechanism underlying how cells preferably undergo anti-inflammatory response remained unclear. Indeed, we observed an increase of iNOS expression, which was typically considered as a pro-inflammatory response of p38 MAPK activation, in MDSCs from PIWIL1-overexpressing cells. It may be possible that the p38 MAPK-induced anti-inflammatory response overrode the pro-inflammatory signaling in MDSCs through negative feedback mechanisms, including IL10 expression.^67^

The role of FAO in regulating the immunosuppressive activities of several immune populations in the cancer microenvironment has been previously studied. FAO activation in dendritic cells and myeloid suppressive cells can foster a tumor cell-favoring immune microenvironment by improving theirs on T-cell inhibitory activities.^53,54,68^ FAO activation may also induce a phenotypic shift of tumor-associated macrophages towards the immunosuppressive M2 phenotype.^69^ Besides, FAO may direct the differentiation of T cells into the suppressive Treg phenotype. These studies indicated that FAO activation in the immune population of the microenvironment can directly regulate cancer immunity during tumor progression.^47,70^ Recently, the role of FAO in cancer cells in the modulation of cancer immunity has also caught some attention. A chemotherapeutic agent 5-fluorouracil induced growth differentiation factor 15 (GDF15) to exacerbate FAO in gastric tumor cells, which promoted M2 differentiation of tumor-associated macrophages in the microenvironment.^71^ Suppression of FAO in liver tumor cells using high-dose dexamethasone activated M1-like tumor-associated macrophages to delay tumor cell growth.^72^ In our study, we observed that activation of FAO in liver cancer cells promoted the immunosuppressive MDSCs populations. The role of complement C3 in the crosstalk between FAO and cancer immunity was never reported. In our study, we found that FAO induced by PIWIL1 overexpression can significantly increase the mitochondrial ROS production that led to oxidative stress. It was previously showed that oxidative stress in the cells is one of the mechanisms of Complement C3 activation.^46^ In this case, we used a mitochondrial ROS scavenger, catalase, to relieve the oxidative stress. Presence of catalase in PIWIL1-overexpressing HCC cells could significantly abolished Complement C3 expression, which indicated that FAO-mediated ROS production is at least partially, if not all, involved as a potential mechanisms of Complement C3 activation in PIWIL1-overexpressing HCC cells.

It was ever extensively reported that FAO is an essential cellular metabolism as an energy provider that mediates the acceleration tumor cell proliferation,^49,50,73^ which was complied with our observation that inhibition of FAO by etomoxir can reduce cellular ATP even in wild type HCC cells. The reduced energy availability in etomoxir-treated HCC cells may reduce the growth rate of HCC cells regardless of PIWIL1 overexpression.^50,74^ Some previous studies have suggested, besides its inhibition on CPT1, that may have a few off-target effects that may lead to tumor inhibition.^28,75^ This may also cause tumor inhibition in both wild type and PIWIL1-overexpressing HCC cells. These factors may, therefore, lead to a tumor inhibition at the same degree in wild type HCC compared with PIWIL1-overexpressing HCC. Indeed, we used etomoxir as a CPT1 inhibitor to repress the elevated FAO in PIWIL1-overexpressing HCC cells. CPT1 is an essential and speed-limiting enzyme in the FAO process, which can be completely blocked by etomoxir. Although we were also looking for a more specific target of PIWIL1 in FAO, from our RNA-seq results it shows that PIWIL1 may regulate several FAO-related genes. In this case, even though we cannot fully conclude that the blockade of tumor growth of PIWIL1-overexpressing HCC by etomoxir was full because of its inhibition on FAO, the observation that the completed blockade of FAO in PIWIL1-overexpressing HCC using etomoxir lead to a tumor with the same degree with that of wild type HCC indicated that blunting FAO can antagonize the tumor-promoting activity of PIWIL1. Furthermore, the presence of etomoxir in HCC cells blunted the regulatory effect of PIWIL1 on immunosuppression-related gene expression, which is the major findings of this study to be reported. With these concerns, we considered the use of etomoxir in the experiments may be sufficient to support our claims to the hypothesis of this study. A previous study suggests in breast cancer cells, a lower dose of etomoxir may be sufficient to suppress FAO without cause cell proliferation.^75^ It may suggest that the FAO process may be irrelevant to cell proliferation in some types of cancer cells. However, some other studies may also suggest that in HCC FAO is an essential cellular metabolism as an energy provider that mediates the acceleration tumor cell proliferation,^49,50,73^ which was complied with our observation that inhibition of FAO by etomoxir can reduce cellular ATP even in wild type HCC cells. In this case, it is a bit difficult for us to identify the proliferation-related and proliferation-unrelated FAO inhibition by etomoxir in HCC. Further studies of dose optimization may be necessary for better data interpretation.

As PMN-MDSCs are naturally present in HCC tumors formed by wild type cells, regardless of PIWIL1 expression, which may also possess tumor-promoting effect, the complete removal of PMN-MDSCs by the injection of anti-Ly6G antibody in HCC tumors formed by wild type cells can also lead to tumor shrinkage. This effect of the anti-Ly6G antibody was also observed in some studies with other types of cancers.^76,77^ This property of the anti-Ly6G antibody may somehow compromise the data interpretation, because from the results we may conclude that PMN-MDSCs are an essential requirement for tumor promotion by PIWIL1, but whether it is a sufficient requirement is still unknown. This antibody has been still extensively used in MDSCs studies though a better solution to make data interpretation accurate is still on the way in many investigations.

## Conclusion

To sum up, in this study, we reported that PIWIL1 plays a vital role in regulating the crosstalk of the metabolic and immune systems of HCC. PIWIL1 overexpression significantly promoted the in vitro proliferation and in vivo growth of HCC, while PIWIL1 knockdown has the opposite effects. This could be due to the increased intracellular ATP production, which could be due to the utilization of fatty acid as an energy source. Suppression of FAO attenuated PIWIL1-induced HCC progression. RNA sequencing analysis revealed that PIWIL1-induced FAO might alter the immune cells in the tumor microenvironment of HCC. The MDSCs population was found accumulated in the hepatic tissues surrounding HCC tumors by local proliferation and infiltration and was responsible for the PIWIL1-derived immunosuppression. PIWIL1-overexpressing HCC cells triggered the activation of p38 MAPK in MDSCs, which produced anti-inflammatory cytokine IL10 that were responsible for the suppression of T-cell immunity. HCC cells-derived complement C3 is the scaffold molecules that delivered the immunosuppressive signal from PIWIL1-overexpressing HCC cells to MDSCs. Expression of PIWIL1 predicted the poor prognosis of HCC patients, and low expression of PIWIL1 suggested a better over survival in HCC patients with a high C3 level. Our findings identify PIWIL1 as an oncogene driving metabolic reprogramming and immune evasion in HCC.

## Materials and methods

### Animal studies

Protocols of all animal studies were approved by the Committee on the Use of Live Animals in Teaching and Research of the University of Hong Kong. Animal handlings have complied with the international standard of animal care and welfare.

### Xenograft model

To establish the xenograft HCC model, 5-week-old immunodeficient mice were subcutaneously injected 5 × 10^6^ MHCC97L cells with or without PIWIL1 overexpression. Mice were observed for 4 weeks. At the end of the study, mice were sacrificed with overdose pentobarbital (200 mg/kg). The tumor was dissected out for measurement. For measurement of tumor volume, we used the calculation formula for caliper measurement as reported by technical reference.^78^ Tumor volume (*V*) = (tumor width (*W*)^2^ × tumor length (*L*))/2.

### Orthotopic HCC model

The orthotopic HCC cells were constituted as our previous report. In brief, under a small cube of HCC tumor (~1 mm in diameter) of luciferase-tagged MHCC97L cells was implanted into the left lobe of the liver of mice. Mice were kept in humane care for 4 weeks. For measuring the weekly growth of the orthotopic tumor, 30 mg/kg luciferin was intraperitoneally injected, and the bioluminescent signal intensity of mice liver was measured under IVIS Spectrum live animal imager (Perkin-Elmer, Germany). At the end of the study, mice were sacrificed with overdose pentobarbital (200 mg/kg). The liver was dissected out for measurement.

### RNA sequencing

Total RNA of vector-expressing and PIWIL1-overexpressing PLC/PRF/5 cells was collected using Trizol reagent (Life Technologies, USA). Then samples were sent to the agented company (Novogene Co., Ltd.) for transcriptomic analysis. The mRNA was first fragmented randomly by the addition of the fragmentation buffer. The library was then constructed and fed into Illumina machines for image acquisition and base calls. Data were then mapped to the reference genome, and differentiated gene expression was analyzed.

### Fatty acid metabolite analysis

Vector- and PIWIL1-overexpressing HCC tissues were homogenized in PBS buffer, and fatty acid metabolites were extracted by methanol, and samples were evaporated to dryness in a vacuum. For the detection of C2 fatty acid, the acetate assay kit (Abcam, UK) was used to quantify intracellular acetate as a C2 representative. For detection of C4-C16, derivatization of the fatty acid metabolites was performed with methoxylamine hydrochloride (15 mg/mL in pyridine) at 70 °C for 60 min, followed by 50 µl N-methyl-N (trimethylsilyl) trifluoroacetamide (MSTFA) with 1% TMCS at 40 °C for 30 min. Samples were then analyzed by GC-MS (Agilent, 6890N-GC with 5973-MS ECD/NPD).

### Statistical analysis

Data was presented in mean ± SEM. Statistical analysis was conducted on one-way ANOVA.

## Supplementary information

Supplmental Files

## Data Availability

The datasets generated during and/or analyzed during the current study are available from the corresponding author on reasonable request.
