# Supplementary material for: PIWIL1 governs the crosstalk of cancer cell metabolism and immunosuppressive microenvironment in hepatocellular carcinoma
Source: Signal Transduct Target Ther. 2021 Feb 26;6:86. doi: 10.1038/s41392-021-00485-8 (PMC7907082; doi:10.1038/s41392-021-00485-8)
Supplement: Supplementary file 1 — Supplmental Files [file 41392_2021_485_MOESM1_ESM.docx]

**Supplementary Materials for**

**PIWIL1 governs the crosstalk of cancer cell metabolism and immunosuppressive microenvironment in hepatocellular carcinoma**

Ning Wang, Hor-Yue Tan, Yuanjun Lu, Yau-Tuen Chan, Di Wang, Wei Guo, Yu Xu, Cheng Zhang, Feiyu Chen, Guoyi Tang, Yibin Feng

**Correspondence to: yfeng@hku.hk**

**This PDF file includes:**

**Materials and Methods**

**Figures. S1 to S6**

**Tables S1**

**Materials and Methods**

***Chemicals, plasmids, and antibodies***

L-arginine, aminoguanidine, etomoxir, SB203580 and SP600125 were purchased from Sigma-Alrich (USA). Vectors alone or expressing human PIWIL1 ORF, shRNA against human PIWIL1, and shRNA against human complement C3 were purchased from Origene (USA). siRNA against MLYCD was purchased from GenePharma (Shanghai, China). Antibody against PIWIL1 and neutralising antibody against C3 were purchased from Abcam (U.K.); antibodies against phosphor-p38 MAPK, p38 MAPK and β-actin were purchased from Cell Signalling Technologies (USA); antibodies against CD11b, Ly6C, Ly6G, F4/80, B220, CD11c, NK1.1, CD3, CD8, Ki67 and Granzyme B were purchased from eBioscience; antibody against Ly6G for in vivo injected was purchase from BioXCells (USA).

***Cell line and cell culture***

Human hepatocellular carcinoma cells PLC/PRF/5 purchased from ATCC (USA). Human hepatocellular carcinoma cells MHCC97L were kindly gifted by Prof. Man Kwan in Department of Surgery, The University of Hong Kong. All the cells were maintained with High Glucose Dulbecco’s Modified Eagle Medium (DMEM) supplemented with 10% Fetal Bovine Serum (FBS) and 1% penicillin/streptomycin (Life Technologies, USA) in a humidified condition (37℃, 5%CO_2_).

***Cell assays***

Cell proliferation assay 1×10^5^ HCC cells were seeded in 24-well plate and culture for 4 days. At day 1,2 and 4 cells were trypsinised, and cell number was counted by hemocytometer.

Colony formation assay 1×10^5^ HCC cells were seeded in 24-well plate and culture for 12 days. Cells were then fixed with 4% paraformaldehyde (PFA) in PBS for 4 h. And 2% Crystal Violet was used to stain the cell colony for 2 h follow by washed. The colony of HCC cells at the size exceeding 1 mm in diameter was counted.

BrdU incorpotation assay 10μM BrdU (BD Pharmingen, USA) was added to the cells and incubated for 5 h at 37℃, 5%CO_2_. Cells were then trypsinised for cell surface staining follow by fixing and permeabilisation on ice for 1 h. Cells were then treated with 30μg DNase to expose incorporated BrdU. FITC-tagged anti-BrdU antibody was added to stain the cells at room temperature for 30 min followed by wash. The incorporation of BrdU was then analysed by flow cytometry.

Cell migration assay 2×10^5^ cells were seeded onto the inserts of Transwell (8.0μm pore size, Corning, USA) in 150μl serum-free medium. 650μl full medium was added to the receiving chamber and allow the cells to moving towards for 3 h. The cells migrated to the basal side of the inserts were then fixed in 4% PFA for 4 h and stained by 2% Crystal Violet. Numbers of cells were counted under a light microscope. 5 scopes per samples were examined.

***Metabolic assay***

Glucose uptake assay The 2-DG glucose uptake assay was performed according to the manufacturer’s instruction (Biovision, USA). In brief, 2000 cells were starved for glucose by preincubating with 100µl Krebs-Ringer-Phosphate-HEPES (KRPH) buffer containing 2 % BSA for 40 min. 10µl of 10 mM 2-DG was added and the cells incubated for 20 min. 10µl Reaction Mix A (8µl Assay buffer, 2µl Enzyme mix) were added into each well and incubated at 37°C for 1 h. unused NAD was then degraded by heating at 90°C for 40 min. 38 µl Reaction Mix B (20µl Glutathione Reductase I, 16µl Substrate (DTNB), 2µl Recycling mix) into each well. Measurement was made at 412 nm in a microplate reader at 37°C every 5 minutes.

Intracellular ATP measurement Intracellular ATP was measured by a commercial kit (Biovision, USA). In brief, cells were lysed with ATP assay buffer and deproteinise using 10 kDa spin column. 50μl samples were then mixed with reaction master mix (44μl ATP assay buffer, 2μl ATP probe, 2μl ATP converter and 2μl developer) and incubated for 30min at room temperature. The absorbance was measured at 570nm using a microplate reader (Labsystems, Finland).

ECAR and OCR assay ECAR and OCR of HCC cells were measured on X.F. Extracellular Flux analyser (Seahorse Bioscience, USA). 1.5×10^6^ cells were suspended in X.F. assay medium and re-adhere for 30 min in a CO_2_-free incubator at 37℃. Measurement of OCR and ECAR was done at baseline and sequential injections of 1μM oligomycin (Sigma-Alrich, USA), 1.5μM FCCP (Sigma-Alrich, USA), and 10μM antimycin A (Sigma-Alrich, USA). The 12-measurement regime was applied, and each measurement was taken every 10min. To calculate the basal respiration, the mean of measurement 2 and 3 were taken; to calculate the maximal respiration, the mean of measurement 7-9 was taken. The OCR: ECAR ratio at basal and maximal respiration was calculated.

Lactate measurement The extracellular and intracellular lactate were measured by a commercial kit (Biovision, USA). 50μl of samples were mixed with reaction master buffer (46μl Lactate assay buffer, 2μl lactate enzyme mix, 2μl probe) and incubated for 30min at room temperature. The absorbance was measured at 570nm using a microplate reader (Labsystems, Finland).

Oleate FAO assay The FAO assay was performed under the manufacturer’s instruction (Abcam, U.K.). In brief, 6×10^4^ cells were cultured in glucose-free medium overnight. 90μl F.A. measurement media (0.5mM L-carnitine, 2.5mM glucose, 150μM FAO oleate-conjugate) and 10μl extracellular O2 consumption reagent were added, and the reaction well was sealed with100μl high sensitivity mineral oil. The samples were then read under fluorescence microplate reader every 5min for totally 60min.

ROS measurement Cells were stained with 50μM DCFH-DA (Sigma-Aldrich, USA) in the dark for 30 min in room temperature. After wash, cells were be analysed by flow cytometry. The intensity of FITC signal suggested the intracellular ROS level.

Free fatty acid measurement Cells were extracted by homogenisation with 200µl of1% Triton X-100 in pure chloroform. The intracellular free fatty acid was measured using a commercial kit (Biovision, USA). In brief, the cell extract was mixed with 2μl Acyl-CoA reagents and 50μl reaction mix (44μl assay buffer, 2μl fatty acid probe, 2μl enzyme mix, 2μl enhancer), and incubated for 39min at 37℃. The absorbance was measured at 570nm using a microplate reader (Labsystems, Finland).

***Fluorescence-activated cell sorting (FACS)***

For collecting the MDSCs from hepatic tissues, the liver of mice was dissected out, and hepatic tissues surrounding HCC tumour (5 mm distance) were obtained and minced. The tissues were then digested in 0.8 mg/mL collagenase IV at 37℃ for 40 min with gentle shaking. Then the supernatant was then collected and filtered through 70μm cell strainer. Pellets were then centrifuged down and resuspended in 36% Percoll (G.E. Healthcare, Germany). Pellets collected from centrifugation was then subject to cell surface staining and the CD11b+Ly6G+Ly6C- population was collected by B.D. Influx cell sorter (B.D. Bioscience, USA). For collecting the T lymphocytes, mice spleen was minced. Splenocytes were then subject to ACK (BD Pharmingen, USA) to lyse the red blood cells, followed by cell surface staining. The CD3+CD8+ cytotoxic T lymphocytes were collected by B.D. Influx cell sorter (B.D. Bioscience, USA).

***BMDM isolation***

Bone marrow cells were flushed from the femur of the mice. After filtered through 70μm cell strainer, 7 mL of cell suspension was carefully added to the top of 3 mL ficoll reagent (Sigma-Aldrich, USA). Cells were centrifuged at 2300 rpm for 20 min, unbrake. Cells at the interface layer of two separated phases were collected and subject to indicated induction.

***In vivo proliferation and migration assays***

For studying the local proliferation of PMN-MDSCs, Edu was injected intraperitoneally into mice at the dose of 10μg/g B.W. 5 h before sacrifice. Hepatic tissue surrounding HCC tumour was removed and digested to isolate the cells. The Click-iT Edu assay was then performed according to the manufacturer’s instruction (Lifetechnologies, USA). For the study of the migration of PMN-MDSCs, 1×10^7^ freshly isolated BMDMs were stained with 2.5μM PKH26PCL dye (Sigma-Aldrich, USA) for 30 min at room temperature. After washing the available dyes, the cells were intraperitoneally injected to the mouse 48 h before sacrifice. Hepatic tissue surrounding HCC tumour was removed and digested to isolate the cells. CD11b+Ly6G+Ly6C- cells were gated to analyse the proliferation and infiltration of PMN-MDSCs in vivo.

***T cell suppression assay***

5×10^5^ sorted CD3+CD8+ cytotoxic T cells were seeded into the 96-well plates coated with 10μg/mL anti-CD3e antibody (eBioscience, USA). The same amount of PMN-MDSCs cells were added with 2μg/mL anti-CD28 antibody (eBioscience, USA) was supplemented for T cell stimulation. After 72-h incubation, CD3+CD8+ cells were fixed and permeabilised on ice for 1 h. And antibody against Ki67 (eBioscience, USA) or Granzyme B (eBiosicence, USA) was added to stain corresponding intracellular protein for 30 min. The expression of proteins was then analysed by flow cytometry.

***qRT-PCR***

Total RNA was isolated with Trizol reagent (Life Technologies, USA), and the mRNA were reverse transcribed with first-strand cDNA synthesis kit (Takara, Japan). Expression of mRNA transcripts was analysed with Light Cycler 480 Quantitative Real-Time PCR (Roche, USA) using the SYBR Green reagent (Takara, Japan). The primer sequences were shown in Table S1.

***Immunoblotting***

Proteins were separated on sodium dodecyl sulfate-polyacrylamide gel electrophoresis (SDS-PAGE) and transferred to a polyvinylidene fluoride membrane (PVDF, Biorad, USA) and blocked with 5% BSA in buffer containing Tris (10 mmol/L, pH 7.4), NaCl (150 mmol/L), and Tween 20 (1%) (TBST followed by incubation with primary antibody overnight at 4℃. The membrane was then subject to the secondary antibody, and the immune-reactivity was detected by chemiluminescence imaging (Biorad) with ECL select kit (G.E. Healthcare, U.K.).

***ELISA assay***

Expression of IL10 and C3 were determined under the manufacturer’s instruction. All experiments were conducted in triplicate. Briefly, samples were added to the antibody-coated wells for 2 h incubation at room temperature and washed. This was followed by incubation of biotinylated antibody for 1 h at room temperature. Plates were then washed and streptavidin antibody was added for 1 h incubation at room temperature. Substrate solution was then added for 30 min incubation in the dark at room temperature. Stop solution was added, and the absorbance at 450 nm was read on Multiskan MS microplate reader (Labsystems, Finland).

**Supplemental Figures**


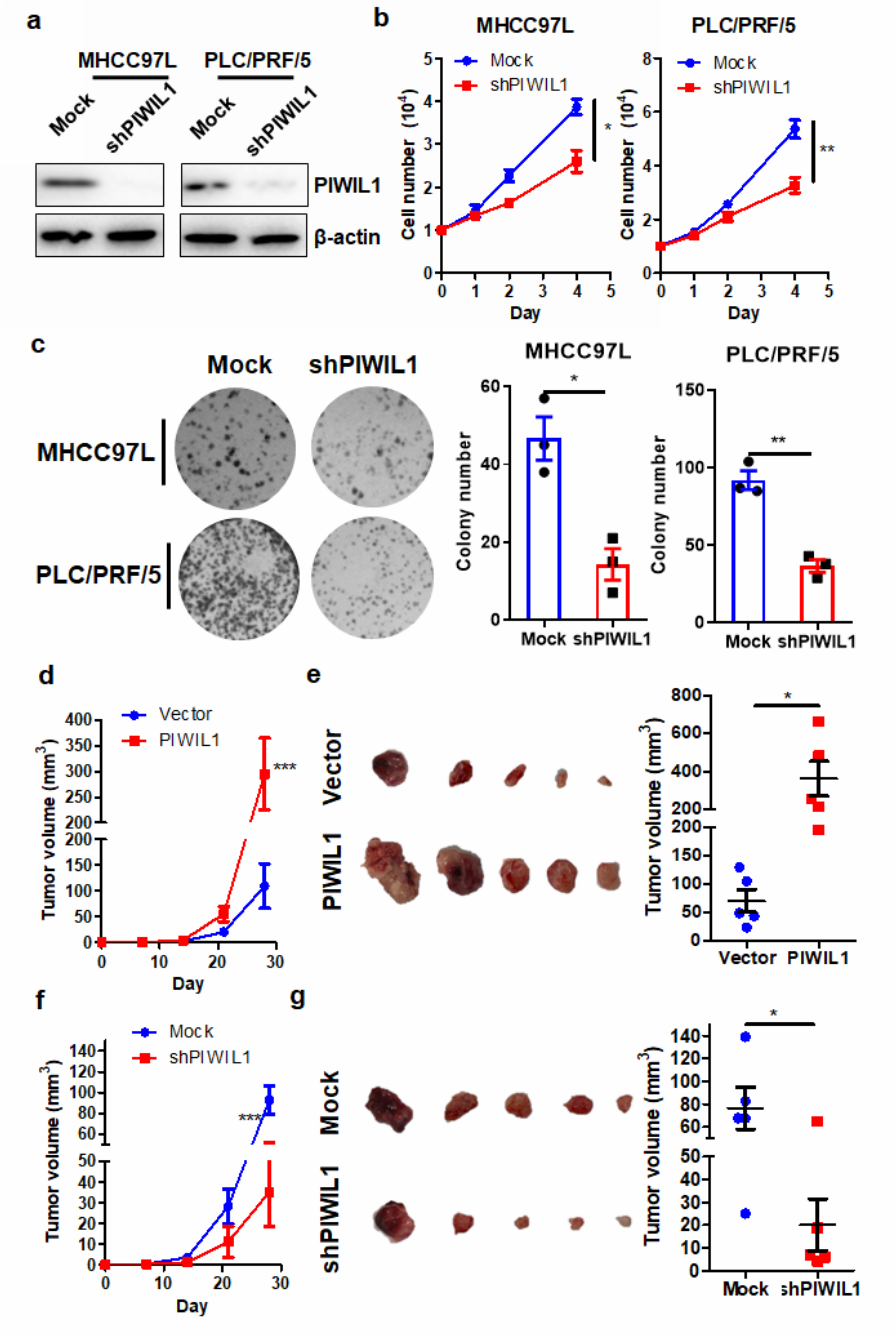


**Figure S1 Knockdown of PIWIL1 reduced in vitro proliferation and in vivo growth of HCC. a.** Stable knockdown clones of MHCC97L and PLC/PRF/5 were constructed by transfection of shRNA against PIWIL1. The selection was made with G418 (50μg/mL). Expression of PIWIL1 was verified by immunoblotting; Knockdown of PIWIL1 in HCC cells significantly reduced the **b.** proliferation rate and **c. the** ability to form a colony; Overexpression of PIWIL1 could significantly induce **d.** the xenograft growth and **e.** size of HCC tumour (n=5); while knockdown of PIWIL1 appeared to suppress the **f.** rapid growth and **g.** size of HCC in a xenograft model (n=5). All experiments were performed in triplicate if without particular notice. *p<0.05; **p<0.01; ***p<0.001.


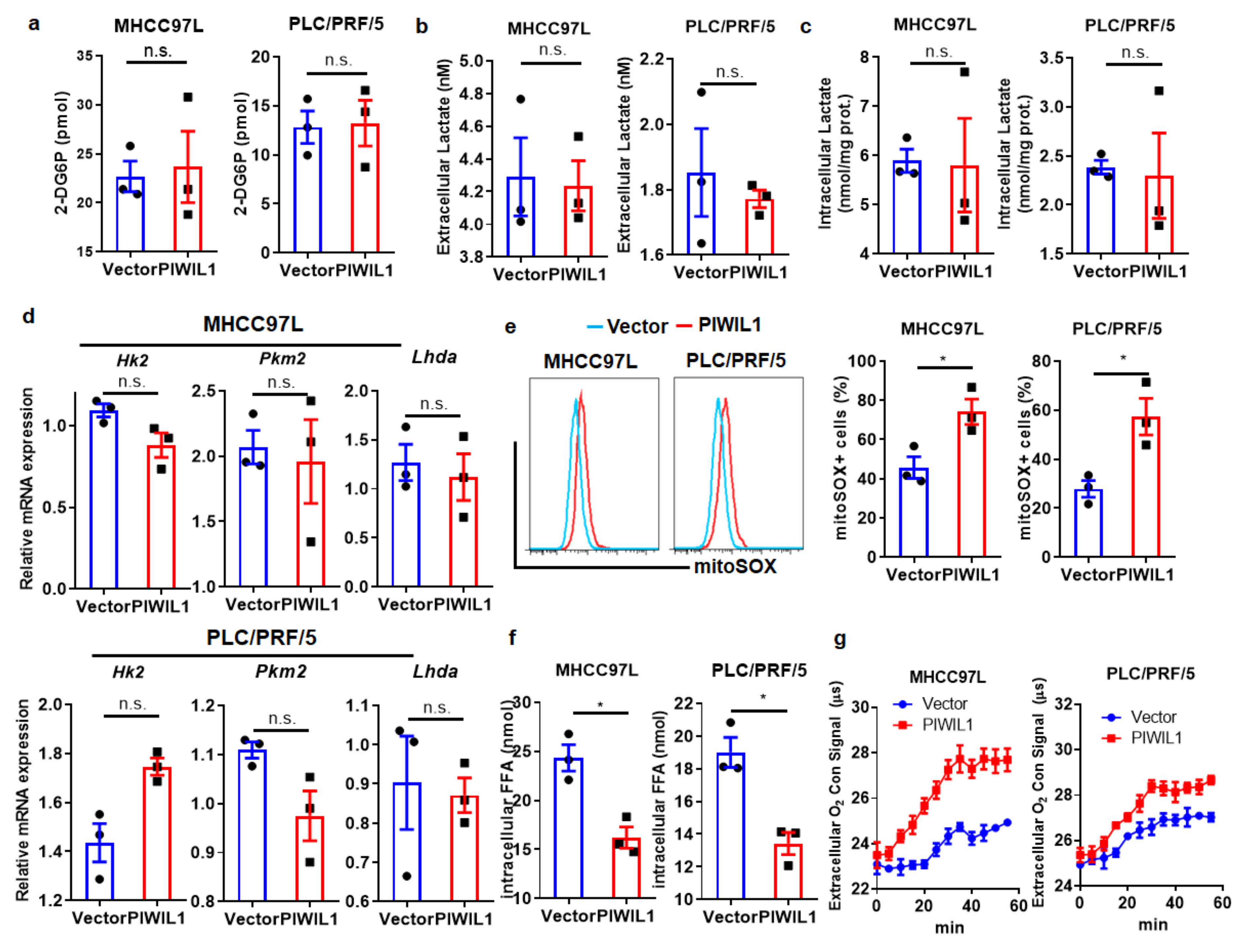


**Figure S2 PIWIL1 regulates energy metabolisms of HCC cells. a.** Overexpression of PIWIL1 did not increase the glucose uptake of HCC cells as indicated by 2-DG uptake assay; Overexpression of PIWIL1 did not significantly change the extracellular **(b)** or intracellular **(c)** levels of lactate; **d.** Expression of glycolytic genes was examined by qRT-PCR, but PIWIL1 overexpression appeared to have minimal effect on related gene expression; **e.** mitoSOX Red staining suggested that PIWIL1 overexpression significant increased the mitochondrial ROS level; **f.** Overexpression of PIWIL1 reduced the intracellular level of free fatty acid in HCC cells; **g.** Overexpression of PIWIL1 could potently increase the consumption of oleate by β-oxidation in HCC cells. All experiments were performed in triplicate. *p<0.05.


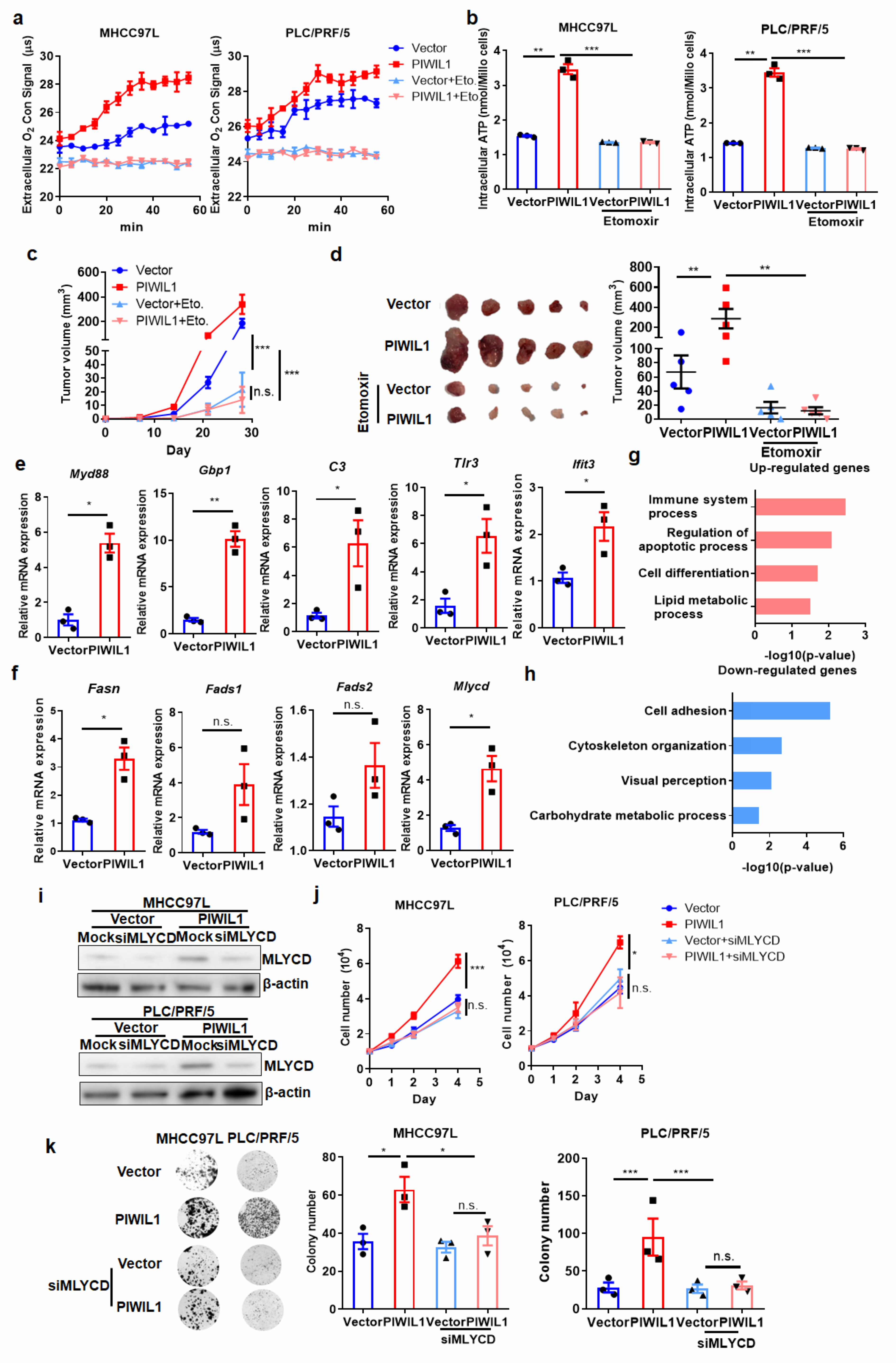


**Figure S3 PIWIL1-facilitated FAO is responsible for HCC cell proliferation and tumour growth.** Wildtype and PIWIL1-overexpressing HCC cells were tested for the ability of oleate β-oxidation in the presence of 100μM etomoxir. Presence of etomoxir significantly suppressed the FAO rate of both wildtype and PIWIL1-overexpressing HCC cells **(a)** and reduced the intracellular ATP level **(b)**. Xenograft of wildtype and PIWIL1-overexpressing HCC was established, and mice were treated with etomoxir (50 mg/kg) every other day by intraperitoneal injection. The growth rate **(c)** and tumour size **(d)** of both wildtype and PIWIL1-overexpressing HCC were significantly reduced in the presence of etomoxir (n=5); RNA sequencing analysis was performed to identify the gene expression changes by PIWIL1 overexpression. Changes of expression of selected genes related to immune system **(e)** and fatty acid metabolism **(f)** was validated by qPCR; and biological pathways related to **(g)** up-regulated genes and **(h)** down-regulated genes were enriched by G.O. analysis. Expression of MLYCD was knockdown using siRNA in both HCC cell lines **(i)**, and knockdown of MLYCD could significantly attenuate the PIWIL1-induced HCC cell proliferation **(j)** and colony formation **(k)**. All experiments were performed in triplicate if without particular notice. *p<0.05; **p<0.01; ***p<0.001.


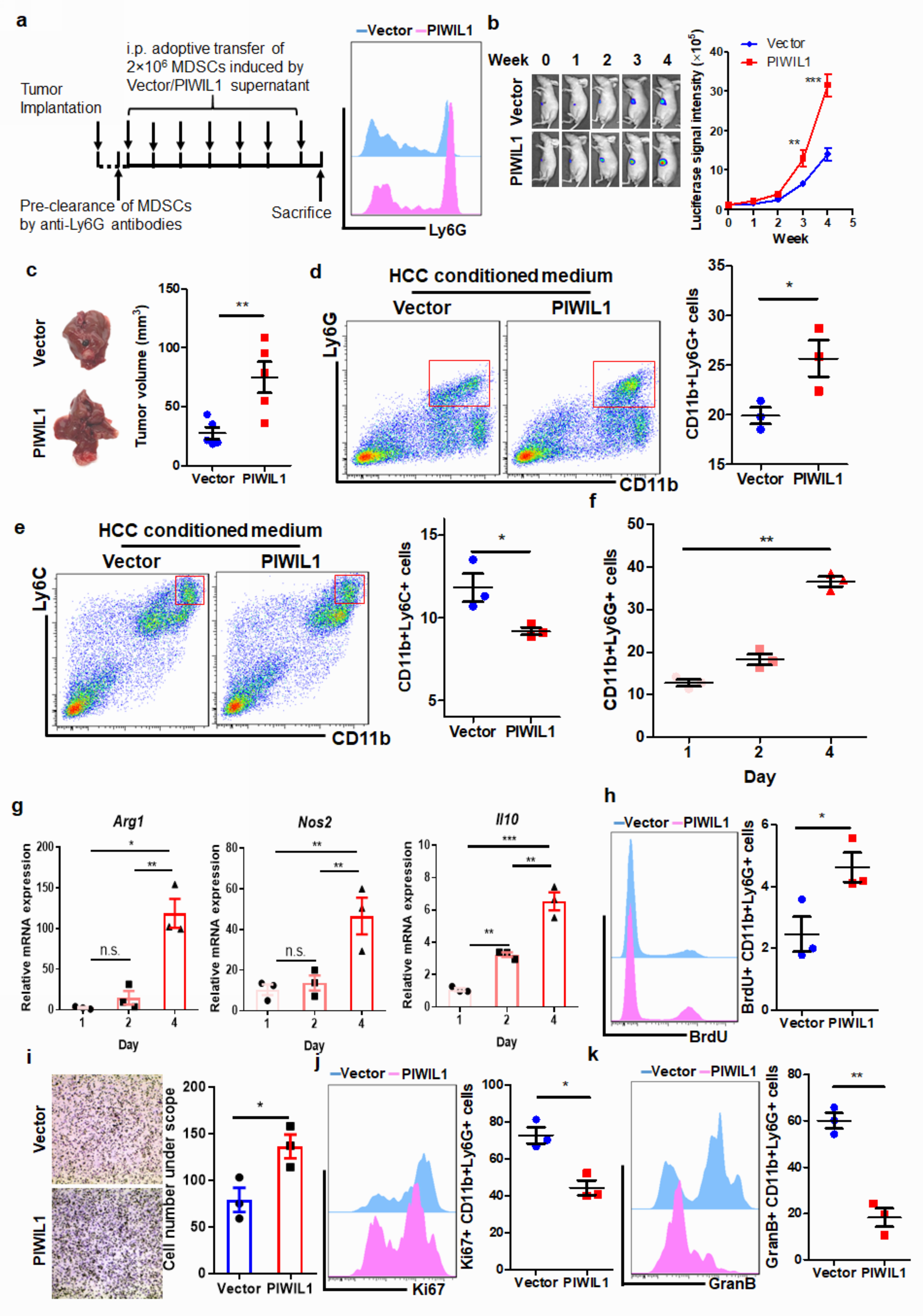


**Figure S4 PIWIL1-overexpressing HCC fosters immunosuppressive activity of MDSCs.** **a.** Schematic protocol for MDSCs adoptive transfer in orthtotopic HCC mice. Orthotopic tumor of mice receiving adoptive transfer of MDSCs cultured with PIWIL1-overexpressing HCC cell supernatant exhibited significant faster growth **(b)** and larger size **(c)** than those with MDSCs cultured by vector expressing HCC cell supernatant.The BMDMs were isolated using ficoll method, and cultured with 50% conditioned medium from wildtype or PIWIL1-overexpressing HCC cells for 4 days. BMDMs cultured with conditioned medium from PIWIL1-overexpressing HCC cells showed **(d)** significant induction of CD11b+Ly6G+Ly6C- a population with potent suppression of **(e)** CD11b+Ly6G-Ly6C^hi^ cells; **f.** We analysed the cells are 1-, 2- and 4-day culture, and found that the induction of CD11b+Ly6G+Ly6C- population by conditioned medium from PIWIL1-overexpressing HCC cells was positively correlated with time of incubation; BMDMs cultured with conditioned medium from PIWIL1-overexpressing HCC cells showed increased **(g)** expression of MDSCs-related genes, **(h)** cell proliferation and **(i)** ability of migration; and exhibited a more potent inhibition on the expression of **(j)** Ki67, a marker of expression and **(k)** Granzyme B (GranB), a marker of activation of co-cultured stimulated CD8+ cytotoxic T cells. All experiments were performed in triplicate. *p<0.05; **p<0.01; ***p<0.001.


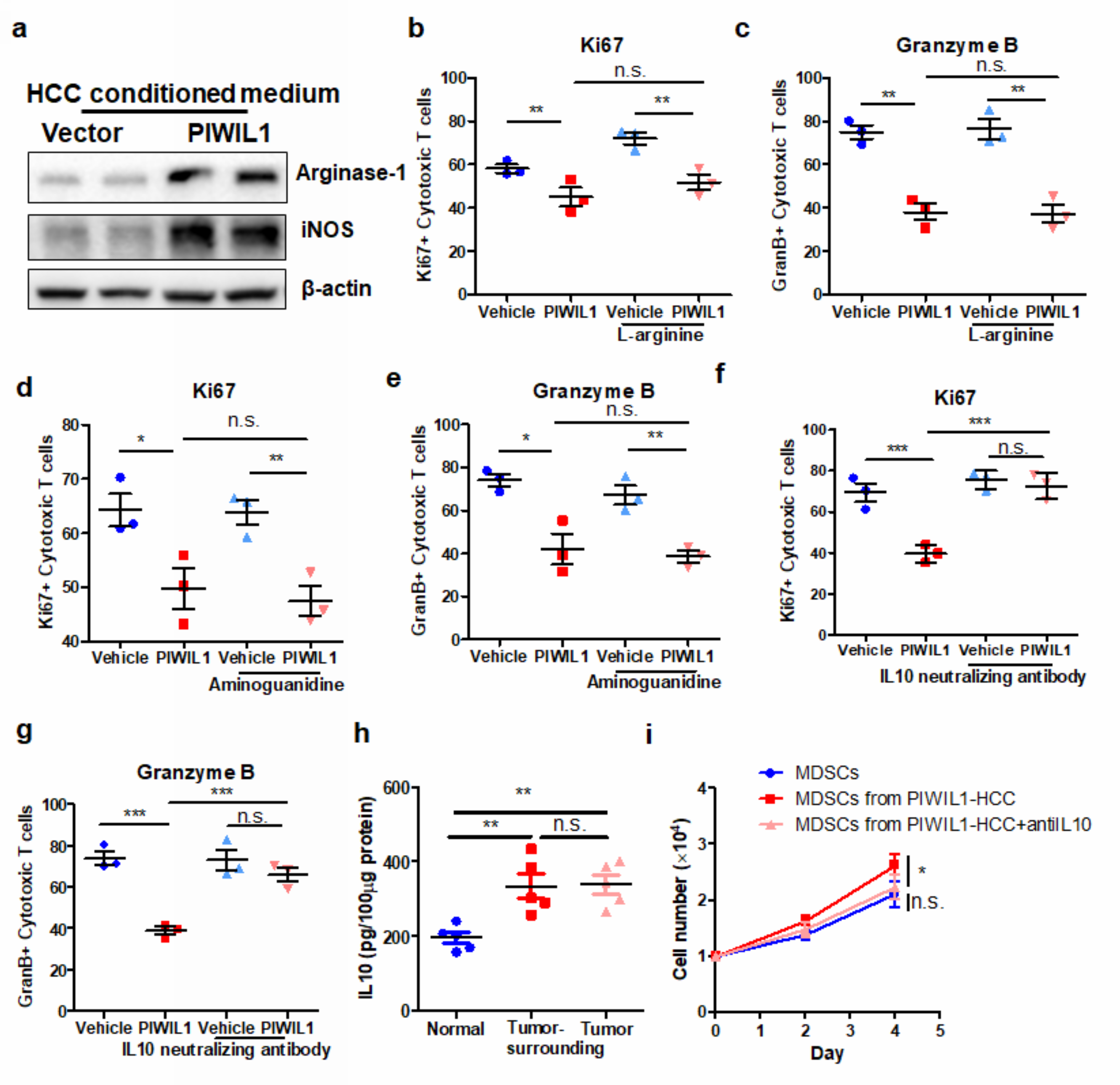


**Figure S5 PIWIL-overexpressing HCC cells stimulate the IL10-associated suppressive activity of MDSCs on T cells. a.** Expression of intracellular arginase-1 and iNOS in sorted PMN-MDSCs from the surrounding hepatic tissue of wildtype and PIWIL1-overexpressing orthotopic HCC was measured by immunoblotting. Cells from PIWIL1-overexpressing HCC showed a significant higher level of expression of arginase-1 and iNOS; The BMDMs were isolated using ficoll method, and cultured with 50% conditioned medium from PIWIL1-overexpressing HCC cells for 4 days. Then cells were co-cultured with stimulated CD8+ cytotoxic T cells in the presence or absence of Arginase-1 substrate L-arginine (L-Arg), iNOS inhibitor aminoguanidine (AGD) or neutralising antibody against IL10. Presence of L-arg or AGD seems showed minimal effect on the inhibition of expression of **(b, d)** Ki67 or **(c, e)** Granzyme B in T cells co-cultured with MDSCs induced by conditioned medium from PIWIL1-overexpressing HCC cells, while IL10 neutralising antibody could potently recover the **(f)** Ki67 and **(g)** Granzyme B expression in these T cells. **h.** The level of IL10 in the tumor and hepatic tissues surrounding the tumors was checked. Although both tumors and the surrounding hepatic tumor was infiltrated with a higher level of IL10 than normal liver tissues, the difference of IL10 level in tumor and its surrounding hepatic tissues is not significant; **i.** the in vitro co-culture study showed that HCC cells co-cultured with sorted wild type and PIWIL1-overexpressing MDSCs exhibited increased proliferation rate, which can be blocked by the presence of neutralizing antibody of IL10. All experiments were performed in triplicate. *p<0.05; **p<0.01; ***p<0.001.


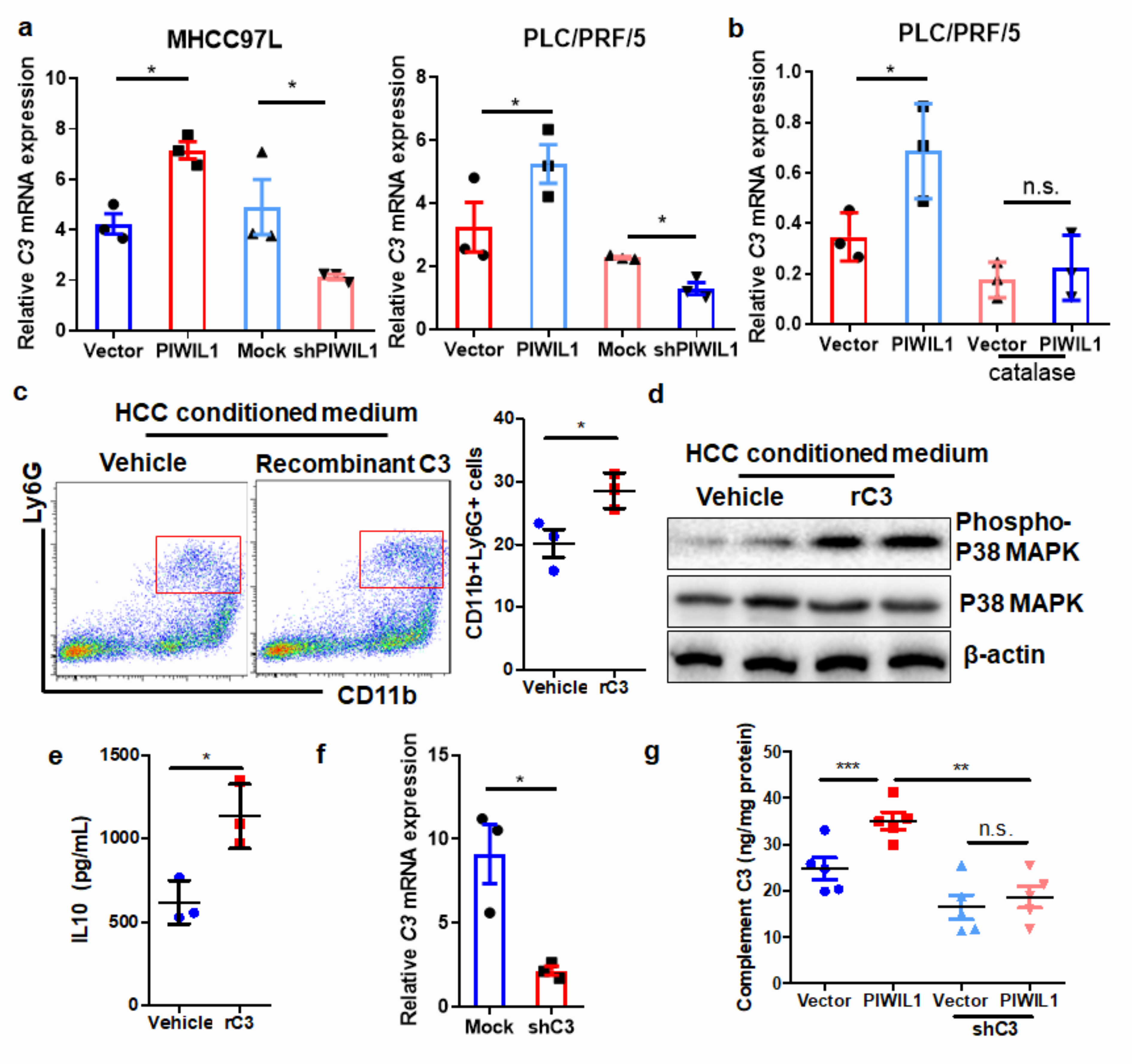


**Figure S6 PIWIL1 induces Complement C3 expression in HCC cells. a.** the mRNA expression of C3 was measured by qRT-PCR. Knockdown of PIWIL1 in HCC cells could significantly reduce the C3 mRNA expression; **b.** Presence of catalase (50 U/mL) attenuated the increased C3 mRNA expression in PIWIL1-overexpressing HCC cells; The BMDMs were isolated using ficoll method, and cultured with 50% conditioned medium from wildtype HCC cells for 4 days in the presence of recombinant human complement C3 protein (100 ng/mL). Supplementation of recombinant C3 protein, **(c)** induced the population of CD11b+Ly6G+Ly6C- cells, **(d)** activated p38 MAPK signalling and **(e)** increased IL10 secretion of cultured MDSCs; **f.** A stable C3 knockdown clone of PIWIL1-overexpressing MHCC97L cells was constructed, and suppression of C3 expression was verified by qRT-PCR. Orthotopic HCC model was established with PIWIL1-overexpressing tumour cells with or without C3 knockdown. **g.** Knockdown of C3 in PIWIL1-overexpressing HCC cells could significantly reduce C3 concentration (n=5). All experiments were performed in triplicate if without particular notice. *p<0.05; **p<0.01; ***p<0.001.

**Supplement Tables**

Table S1 Primers for qRT-PCR analysis

| Gene name | Forward primer | Reverse primer |
| --- | --- | --- |
| Murine Nos2 | CGAAACGCTTCACTTCCAA | TGAGCCTATATTGCTGTGGCT |
| Murine Arg1 | AACACGGCAGTGGCTTTAACC | GGTTTTCATGTGGCGCATTC |
| Murine Il10 | TGGCCCAGAAATCAAGGAGC | CAGCAGACTCAATACACACT |
| Murine Pdl1 | GCATTATATTCACAGCCTGC | CCCTTCAAAAGCTGGTCCTT |
| Murine Tgfb | GACCGCAACAACGCCATCTA | GGCGTATCAGTGGGGGTCAG |
| Murine β-actin | TCTACGAGGGCTATGCTCTCC | GGATGCCACAGGATTCCATAC |
| Human C3 | GCTGCTCCTGCTACTAACCCA | AAAGGCAGTTCCCTCCACTTT |
| Human Pkm2 | TGTCTGGAGAAACAGCCAAG | TCCTCGAATAGCTGCAAGTG |
| Human Ldha | GGTTGGTGCTGTTGGCATGG | TGCCCCAGCCGTGATAATGA |
| Human Myd88 | GACCCCTGGTGCAAGTACC | AGTAGCTTACAACGCATGACAG |
| Human Gbp1 | GTGGAACGTGTGAAAGCTGA | CAACTGGACCCTGTCGTTCT |
| Human Tlr3 | GTATTGCCTGGTTTGTTAATTGG | AAGAGTTCAAAGGGGGCACT |
| Human Ifit3 | TGAGGAAGGGTGGACACAACTGAA | AGGAGAATTCTGGGTTGTTGGGCT |
| Human Fasn | CTTCCGAGATTCCATCCTACGC | TGGCAGTCAGGCTCACAAACG |
| Human Fads1 | AGGAGCGGTGGCTAGTGAT | GGCTGCTCTGGAGACAGTTC |
| Human Fads2 | CCGCAAGGTTTACAACATCA | TCAGCAGGGGTTTCAAGAAC |
| Human Mlycd | TTGCACGTGGCACTGACT | GGATGTTCCTTCACGATTGC |
| Human β-actin | CATGTACGTTGCTATCCAGGC | CTCCTTAATGTCACGCACGAT |
